# Prognostic significance of extracapsular extension in patients with non-small cell lung cancer following neoadjuvant chemoimmunotherapy: a retrospective cohort study

**DOI:** 10.3389/fimmu.2026.1799856

**Published:** 2026-07-15

**Authors:** Jianing Qiu, Shaoling Li, Linfeng Ge, Suyu Wang, Xinlei Hou, Xinyu Ling

**Affiliations:** 1 Department of Thoracic Surgery, Shanghai Pulmonary Hospital, School of Medicine, Tongji University, Shanghai, China; 2 Department of Pathology, Shanghai Pulmonary Hospital, School of Medicine, Tongji University, Shanghai, China; 3 Department of Thoracic Surgery, Daqing Oilfield General Hospital, Daqing, China

**Keywords:** disease-free survival, extracapsular extension, neoadjuvant chemoimmunotherapy, non-small cell lung cancer, overall survival

## Abstract

**Background:**

Non-small cell lung cancer (NSCLC) is a leading cause of oncology-related mortality. Although neoadjuvant chemoimmunotherapy (NCIT) improves pathologic response, disease recurrence remains common. Extracapsular extension (ECE) is recognized as a hallmark of aggressive tumor biology across malignancies; however, its prognostic significance in the NCIT setting remains to be fully elucidated. This study aims to evaluate the impact of ECE on survival outcomes in patients with NSCLC following NCIT.

**Methods:**

A total of 104 patients with NSCLC and pathologically confirmed lymph node metastasis who underwent surgery following NCIT were retrospectively enrolled. Kaplan-Meier analysis and Cox proportional hazards regression evaluated the prognostic impact of ECE. To minimize selection bias, propensity score matching (PSM) analyses were performed. Time-dependent receiver operating characteristic (ROC) curves assessed predictive accuracy.

**Results:**

ECE-positive patients had significantly worse DFS than ECE-negative counterparts; multivariable analysis confirmed that ECE was an independent prognostic factor for DFS (HR = 2.37, 95% CI: 1.28–4.41, P = 0.006). Furthermore, integrating ECE status with major pathologic response (MPR) substantially improved the predictive accuracy for 2-year DFS compared to MPR alone (AUC increased from 0.591 to 0.706). Although ECE did not significantly affect OS, lymphovascular invasion (LVI) emerged as independent predictor of OS (HR = 3.99, 95% CI: 1.59–10.02, P = 0.003).

**Conclusions:**

ECE remains a potent driver of recurrence following NCIT; intensified postoperative surveillance is warranted for these patients to enable early detection and management of relapse.

## Introduction

Non-small cell lung cancer (NSCLC) remains the leading cause of cancer deaths globally, with surgical resection serving as the primary curative intent for patients with early-stage and locally advanced disease (1, 2). Neoadjuvant chemoimmunotherapy (NCIT) has recently emerged as the standard of care for resectable NSCLC, based on pivotal clinical trials demonstrating its efficacy (3–5). Although NCIT has significantly improved pathologic complete response (pCR) and major pathologic response (MPR) rates, a subset of patients still experiences disease recurrence despite achieving favorable pathologic remission, which ultimately compromises long-term survival (4, 6). This underscores the critical need to refine the assessment of residual pathological risk to better identify patients who remain at high risk of recurrence and subsequent mortality following neoadjuvant treatment.

Among various pathological features, nodal status remains a pivotal determinant of prognosis in NSCLC (7). Specifically, extracapsular extension (ECE), defined as the infiltration of tumor cells through the nodal capsule into the perinodal adipose tissue, is recognized as a hallmark of aggressive tumor biology (8). The detrimental impact of ECE on survival outcomes has been robustly established across multiple malignancies, including head and neck, breast, and gastrointestinal cancers (9–11). Acknowledging its clinical significance, the IASLC recently proposed that ECE should be considered an indicator of incomplete resection, further emphasizing its role in risk stratification (12). Furthermore, a large-scale cohort study has confirmed that the survival outcomes of patients with ECE are comparable to those of patients with incomplete resection (13). However, existing validation studies have predominantly excluded patients receiving neoadjuvant treatment. Given that NCIT induces unique pathological changes, involving profound tumor regression and extensive remodeling of the nodal microenvironment, the question of whether ECE retains its prognostic value in this setting remains a critical knowledge gap (14).

Therefore, the present study aims to evaluate the prognostic significance of ECE for disease-free survival (DFS) and overall survival (OS) among patients with NSCLC treated with NCIT followed by surgical resection.

## Methods

### Patient selection and data collection

**Figure 1:**
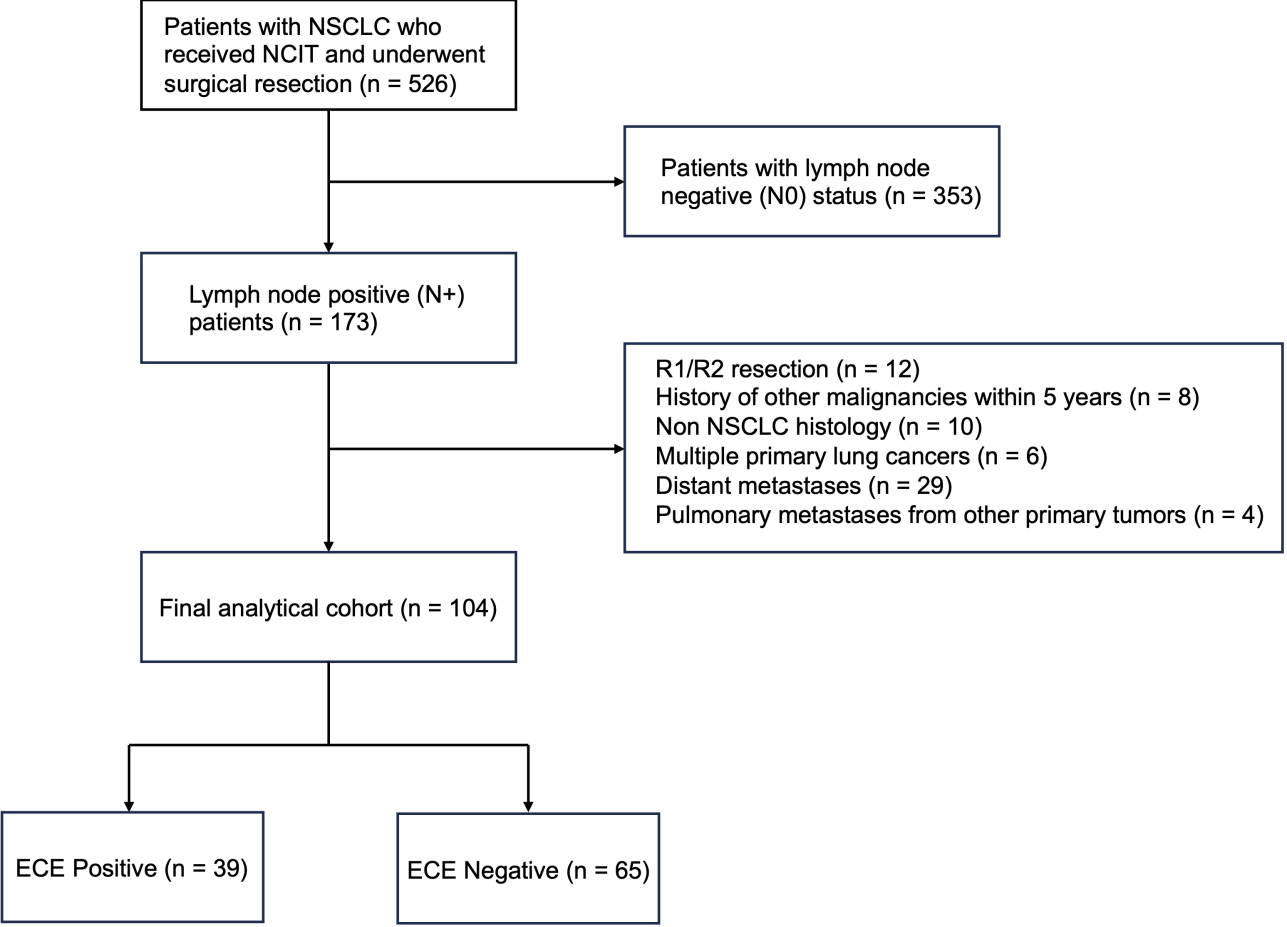
Flow chart.

Patients with NSCLC who underwent surgical resection following PD-1 inhibitor-based NCIT at Shanghai Pulmonary Hospital between 2019 and 2022 were consecutively and retrospectively enrolled in this study. All participants had pathologically confirmed lymph node metastasis. The exclusion criteria were as follows: (i) non-R0 resection; (ii) history of other malignancies within the preceding five years; (iii) histology other than NSCLC; (iv) multiple primary lung cancers; (v) distant metastases identified preoperatively or intraoperatively; (vi) pulmonary metastases from other primary tumors. Ultimately, 104 patients were included in the final analysis (**Figure 1**). This study received approval from the Institutional Review Board of Shanghai Pulmonary Hospital (No. K25-706), with a waiver for informed consent.

Clinical data, including demographics, preoperative imaging, laboratory results, postoperative pathology, and perioperative regimens, were retrieved from electronic medical records. Tumor staging followed the 8th edition of the TNM classification for NSCLC.

### Endpoints and follow-up

Primary endpoints included DFS and OS. DFS was defined as the duration from the surgery to the first instance of tumor recurrence or death. OS was the time interval from the surgery to death from any cause, with surviving patients censored at the last follow-up visit. Survival data were obtained through a combination of electronic medical records and telephone follow-up.

### Pathological assessment of ECE

**Figure 2:**
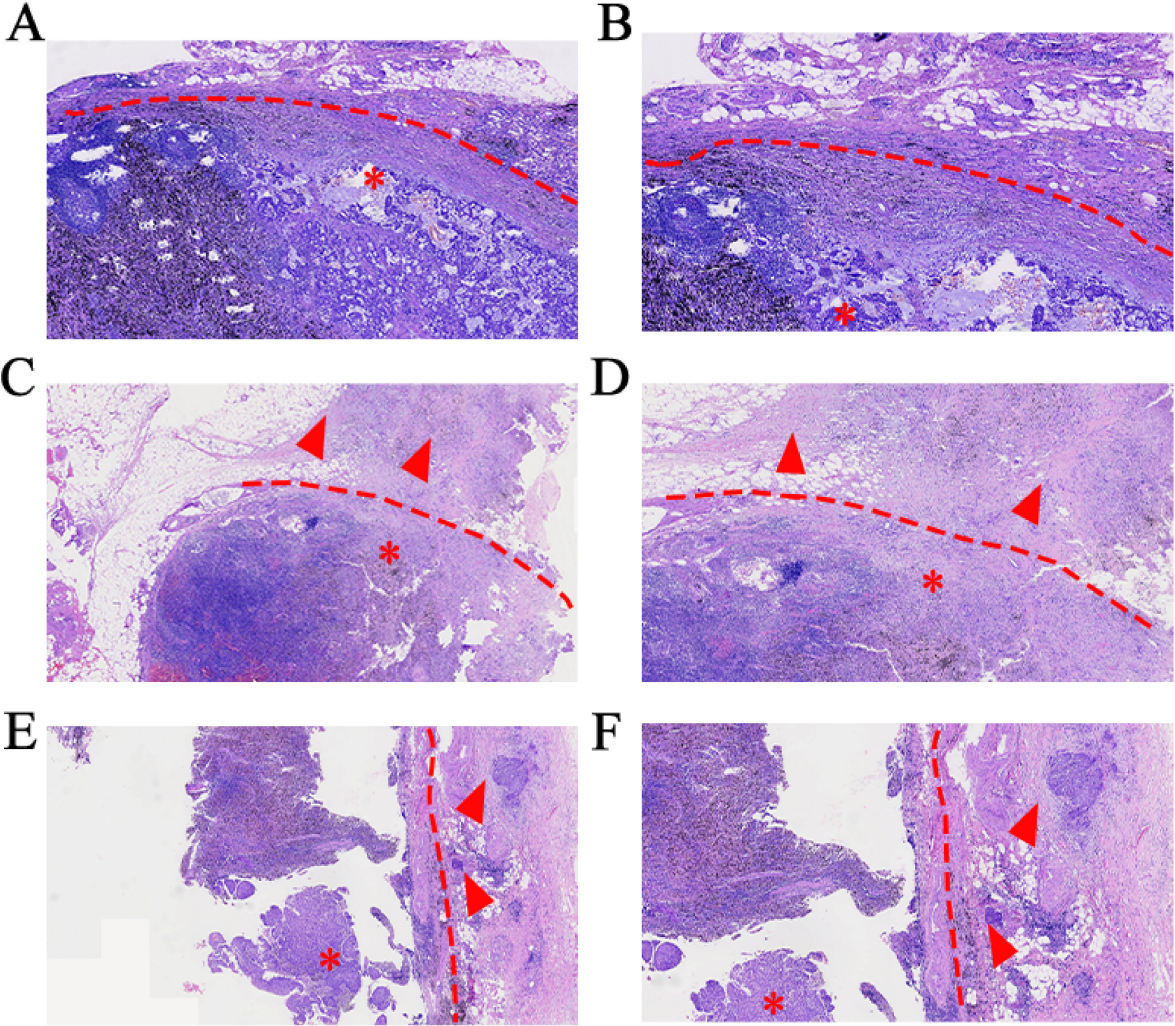
Representative Histopathology of Intranodal and Extranodal Tumor Involvement. Dashed line indicates the lymph node capsule. **(A)** Intranodal tumor involvement: The asterisk denotes tumor tissue within the lymph node (low magnification); **(B)** Intranodal tumor involvement: High magnification view showing a thickened fibrous capsule with tumor cells confined within the node; no capsular breakthrough is observed; **(C)** Extranodal extension (ECE), Low magnification view where the asterisk identifies tumor tissue within the capsule and the triangle represents extranodal tumor deposits; **(D)** ECE, High magnification view demonstrating continuous tumor invasion from the lymph node into the perinodal adipose tissue; **(E)** ECE, Low magnification view showing the distribution of intranodal tumor (asterisk) and extranodal extension (triangle); **(F)** Extranodal extension (ECE), High magnification view showing tumor cells invading the perinodal adipose and fibrocollagenous tissues.

ECE was defined as the infiltration of tumor cells through the lymph node capsule into surrounding perinodal tissues (**Figure 2**). To ensure the diagnostic accuracy, stringent pathological criteria were used to differentiate true ECE from therapy-induced nodal alterations. Treatment-related alterations were typically characterized by specific features, such as aggregated foamy histiocytes and deposited cholesterol clefts. Even in lymph nodes concurrently affected by tumor and neoadjuvant-related changes, the original rounded or oval contour of the node remained clearly discernible. Extracapsular fibrosis resulting from direct tumor extension was predominantly located within the perinodal adipose tissue, extending distinctly beyond the original nodal boundary. In our cohort, cases with fibroblastic ECE typically exhibited spiculated margins and contiguous infiltration into the surrounding adipose tissue. All resected specimens were fixed in 10% buffered formalin and embedded in paraffin for routine hematoxylin and eosin (H&E) staining. The status of ECE were independently evaluated by two senior pathologists (Dr. Li and Dr. Guo) who were blinded to the clinical outcomes. Inter-observer reliability was determined using Cohen’s kappa statistics. As illustrated in **Supplementary Figure 1**, an almost perfect diagnostic consensus was reached between the two pathologists (kappa = 0.835, P < 0.001). Any discrepancies in ECE status or the interpretation of treatment effects were resolved by consensus.

To provide a comprehensive assessment of nodal disease burden, we quantified the total number of resected lymph nodes (LNs), the number of metastatic LNs, and the specific count of LNs exhibiting ECE for each patient. Furthermore, the extent of ECE was stratified into focal or diffuse patterns based on morphological distribution. Focal ECE was defined as the presence of localized metastatic foci infiltrating perinodal tissues. Diffuse ECE was characterized by the complete effacement of the nodal architecture by tumor cells, accompanied by widespread, multidirectional infiltration into the surrounding perinodal environment.

### Neoadjuvant chemoimmunotherapy

Neoadjuvant treatment strategies were formulated via multidisciplinary team (MDT) consultations comprising thoracic surgeons and medical oncologists. The standard regimen typically consisted of an immune checkpoint inhibitor (ICI) administered in combination with platinum-based doublet chemotherapy (predominantly paclitaxel or pemetrexed). Specific ICIs utilized in this study included nivolumab, pembrolizumab, tislelizumab, sintilimab, camrelizumab, toripalimab, and durvalumab.

### Statistical analyses

Categorical variables were summarized as counts and percentages, while continuous variables were presented as medians with interquartile ranges (IQRs). Survival curves for DFS and OS were generated using the Kaplan-Meier method and compared via the log-rank test. Univariate and multivariate Cox proportional hazards regression analyses were performed to identify independent prognostic factors for DFS and OS. Variables showing the strongest associations with survival outcomes in the univariate analyses were selected for inclusion in the multivariate Cox regression models to minimize model overfitting and account for the limited number of events. Hazard ratios (HRs) and 95% confidence intervals (CIs) were estimated. To evaluate the robustness of our primary findings, a sensitivity analysis was performed by including all collected clinicopathological variables in a secondary multivariable Cox model, ensuring that no clinically relevant factors were overlooked due to initial screening thresholds. Furthermore, time-dependent receiver operating characteristic (ROC) curve analyses were employed to evaluate the predictive performance and discriminative accuracy of the identified prognostic factors for survival outcomes over time. The area under the curve (AUC) was calculated to quantify their discriminative ability. To further evaluate the robustness of our findings and reduce potential confounding due to treatment selection bias, propensity score matching (PSM) analyses were performed. Propensity scores were calculated using logistic regression models based on relevant clinicopathological variables. For the analysis of adjuvant therapy efficacy, patients were matched using 1:1 nearest-neighbor matching without replacement, with a caliper width of 0.1. For the prognostic analysis of ECE, 1:2 nearest-neighbor matching without replacement was performed using the same caliper width. After PSM, survival analyses were repeated in the matched cohorts to further evaluate the prognostic impact of ECE and the potential benefit of adjuvant therapy. All analyses were conducted using R software version 4.4.1. Statistical significance was defined as a two-sided P < 0.05.

## Results

### Patient characteristics

A total of 104 patients with NSCLC and confirmed lymph node metastasis were enrolled. The detailed characteristics are summarized in **Table 1**. Patients were predominantly male (91.3%) and smokers (67.3%), with a median age of 63 years (IQR: 56–68). Most patients underwent VATS (n=65, 62.5%) and lobectomy (n=70, 67.3%). Postoperative pathological assessment revealed that 29 patients achieved an MPR. Adjuvant immunotherapy and chemotherapy were administered to 58 (55.8%) and 74 (71.2%) patients, respectively.

**Table 1:** Characteristics of patients with NSCLC stratified by ECE.

Characteristic	Overall N = 104	ECE negative N = 65	ECE positive N = 39	*P* value
Age	63 (56, 68)	64 (57, 68)	62 (54, 67)	0.185
Sex				0.290
male	95 (91.3%)	61 (93.8%)	34 (87.2%)	
female	9 (8.7%)	4 (6.2%)	5 (12.8%)	
Smoking				0.589
No	34 (32.7%)	20 (30.8%)	14 (35.9%)	
Yes	70 (67.3%)	45 (69.2%)	25 (64.1%)	
ECOG				0.386
0	46 (44.2%)	30 (46.2%)	16 (41.0%)	
1	55 (52.9%)	32 (49.2%)	23 (59.0%)	
2	3 (2.9%)	3 (4.6%)	0 (0.0%)	
Histology				**0.010**
Adenocarcinoma	40 (38.5%)	20 (30.8%)	20 (51.3%)	
Squamous cell carcinoma	62 (59.6%)	45 (69.2%)	17 (43.6%)	
Others	2 (1.9%)	0 (0.0%)	2 (5.1%)	
Surgical approach				0.317
Open	28 (26.9%)	20 (30.8%)	8 (20.5%)	
VATS	65 (62.5%)	40 (61.5%)	25 (64.1%)	
RATS	11 (10.6%)	5 (7.7%)	6 (15.4%)	
Surgical type				0.078
Lobectomy	70 (67.3%)	38 (58.5%)	32 (82.1%)	
Bilobectomy	1 (1.0%)	1 (1.5%)	0 (0.0%)	
Sleeve	25 (24.0%)	20 (30.8%)	5 (12.8%)	
Pneumonectomy	8 (7.7%)	6 (9.2%)	2 (5.1%)	
MPR				**0.028**
No	75 (72.1%)	42 (64.6%)	33 (84.6%)	
Yes	29 (27.9%)	23 (35.4%)	6 (15.4%)	
STAS				**0.008**
No	79 (76.0%)	55 (84.6%)	24 (61.5%)	
Yes	25 (24.0%)	10 (15.4%)	15 (38.5%)	
VPI				0.053
No	92 (88.5%)	61 (93.8%)	31 (79.5%)	
Yes	12 (11.5%)	4 (6.2%)	8 (20.5%)	
PNI				0.290
No	101 (97.1%)	62 (95.4%)	39 (100.0%)	
Yes	3 (2.9%)	3 (4.6%)	0 (0.0%)	
LVI				**0.017**
No	73 (70.2%)	51 (78.5%)	22 (56.4%)	
Yes	31 (29.8%)	14 (21.5%)	17 (43.6%)	
ypT				0.279
0	13 (12.5%)	10 (15.4%)	3 (7.7%)	
1	38 (36.5%)	27 (41.5%)	11 (28.2%)	
2	38 (36.5%)	19 (29.2%)	19 (48.7%)	
3	12 (11.5%)	7 (10.8%)	5 (12.8%)	
4	3 (2.9%)	2 (3.1%)	1 (2.6%)	
ypN				0.523
1	36 (34.6%)	24 (36.9%)	12 (30.8%)	
2	68 (65.4%)	41 (63.1%)	27 (69.2%)	
ypStage				0.397
II	29 (27.9%)	20 (30.8%)	9 (23.1%)	
III	75 (72.1%)	45 (69.2%)	30 (76.9%)	
Adjuvant immunotherapy				0.475
No	46 (44.2%)	27 (41.5%)	19 (48.7%)	
Yes	58 (55.8%)	38 (58.5%)	20 (51.3%)	
Adjuvant chemotherapy				0.146
No	30 (28.8%)	22 (33.8%)	8 (20.5%)	
Yes	74 (71.2%)	43 (66.2%)	31 (79.5%)	

ECE, Extracapsular Extension; MPR, major pathologic response; STAS, Spread Through Air Spaces; VPI, Visceral Pleural Invasion; PNI, Perineural Invasion; LVI, Lymphovascular Invasion; ECOG, Eastern Cooperative Oncology Group Performance Status; RATS, robot-assisted thoracic surgery; VATS, video-assisted thoracoscopic surgery. Bold values indicate statistically significant differences (*P* < 0.05).

ECE was identified in 39 (37.5%) patients. It was significantly more frequent in adenocarcinoma (51.3% vs. 30.8%, P = 0.010). Notably, the ECE-positive group exhibited a significantly lower MPR rate compared to the ECE-negative group (15.4% vs. 35.4%, P = 0.028). Furthermore, the presence of ECE was significantly associated with higher incidences of Spread Through Air Spaces (STAS) (38.5% vs. 15.4%, P = 0.008) and Lymphovascular invasion (LVI) (43.6% vs. 21.5%, P = 0.017).

### Prognostic impact of ECE on survival

During the follow-up period (median duration: 35.6 months), a total of 51 recurrences and 19 deaths were documented across the cohort. Specifically, in the ECE-Negative group (n = 65), 25 patients experienced disease recurrence, and 10 died. In comparison, the ECE- Positive group (n = 39) recorded 26 recurrences and 9 deaths. Survival analysis of the overall population showed that the 1-year DFS rate was 75.8% (95% CI: 68.0%–84.5%), and the 2-year DFS rate was 59.8% (95% CI: 51.0%–70.2%).

**Figure 3:**
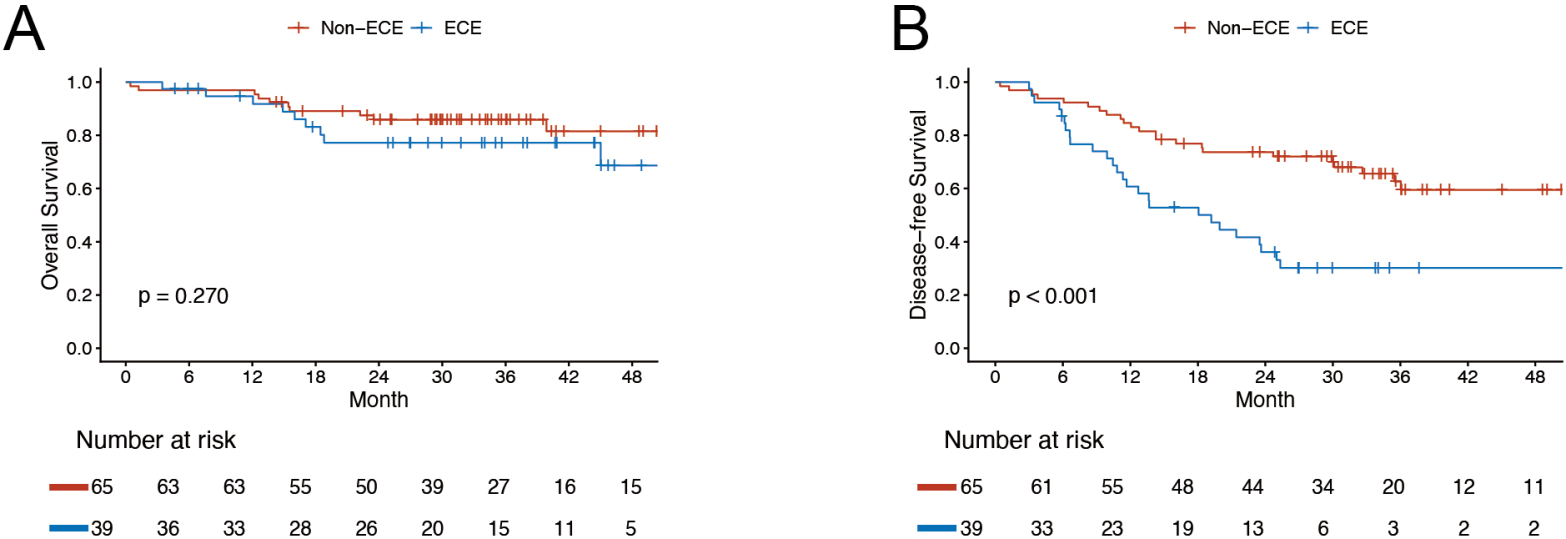
Survival outcomes by ECE status in all patients. **(A)** Overall survival (OS); **(B)** Disease free survival (DFS).

Patients with ECE had significantly shorter DFS than those without (P < 0.001). Although the ECE-positive group exhibited a numerical trend toward poorer OS, the difference was not statistically significant (P = 0.270; **Figure 3**).

**Figure 4:**
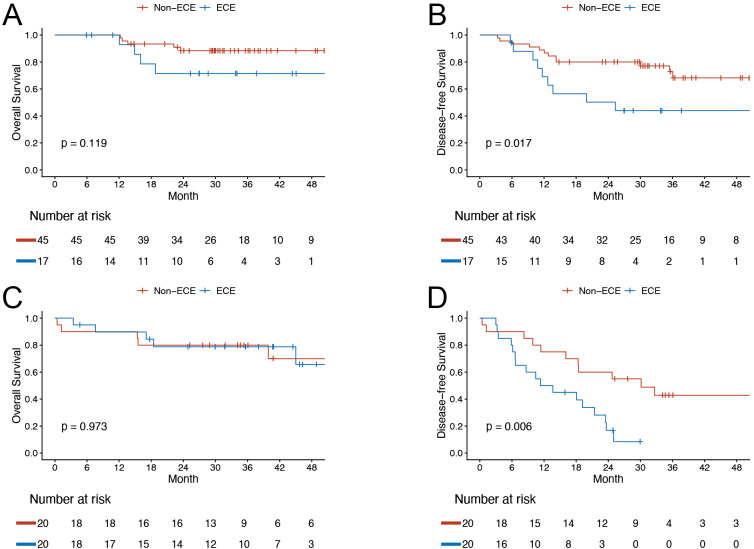
Survival by ECE status and histology. **(A, B)** Squamous cell carcinoma; **(C, D)** Adenocarcinoma. Panels **(A, C)** show OS; **(B, D)** show DFS.

Among adenocarcinoma patients, ECE was significantly associated with shorter DFS (P = 0.006), but not with OS (P = 0.973). In the squamous cell carcinoma subgroup, ECE-positive patients also exhibited significantly poorer DFS (P = 0.017), whereas the association between ECE status and OS did not reach statistical significance (P = 0.119; **Figure 4**).

**Figure 5:**
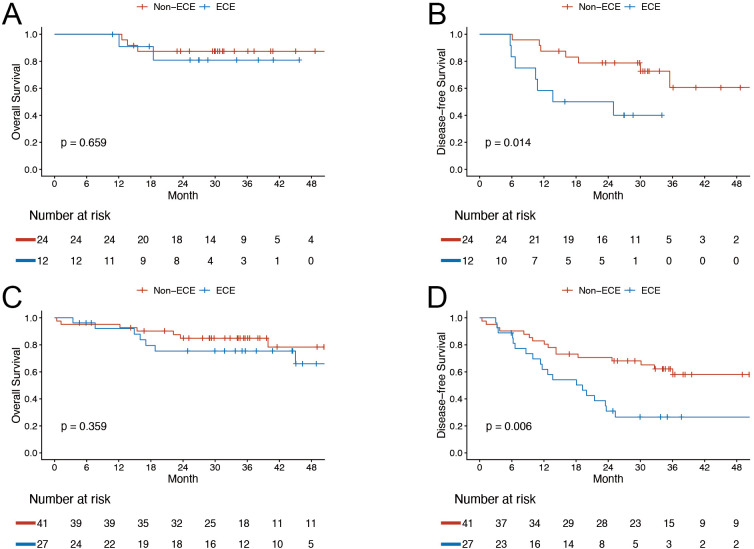
Survival by ECE status and N stage. **(A, B)** N1 stage; **(C, D)** N2 stage. Panels **(A, C)** show OS; **(B, D)** show DFS.

Subgroup analysis based on lymph node status was consistent with the overall cohort. In both N1 and N2 subgroups, the presence of ECE was significantly associated with inferior DFS (N1: P = 0.014; N2: P = 0.006). No significant association was observed between ECE status and OS in either subgroup (N1: P = 0.659; N2: P = 0.359; **Figure 5**).

### Efficacy of adjuvant therapy by ECE status

To evaluate clinical efficacy, the impact of adjuvant therapy was analyzed according to ECE status. In ECE-positive patients, there were no statistically significant differences in OS (P = 0.741) or DFS (P = 0.063) between those who received adjuvant immunotherapy and those who did not. Similarly, in the ECE-negative group, adjuvant immunotherapy did not significantly correlate with improved OS (P = 0.510) or DFS (P = 0.263; **Supplementary Figure 2**). Histological subgroup analysis yielded consistent results. In both adenocarcinoma and squamous cell carcinoma, adjuvant immunotherapy provided no statistically significant survival differences regardless of ECE status (all P > 0.05, **Supplementary Figures 3**, **S4**). Furthermore, adjuvant chemotherapy showed no significant survival impact regardless of ECE status (all P > 0.05, **Supplementary Figures 5**–**S7**).

### Lymph node burden and prognostic impact of ECE patterns

Among the 104 patients analyzed, ECE positivity correlated with an increased metastatic burden. Despite comparable total LNs yields between the ECE-negative and ECE-positive groups, the ECE-positive cohort exhibited a significantly higher metastatic LN count (median 3 [IQR 2, 6] vs. median 2 [IQR 1, 4], P = 0.006, **Supplementary Table 1**). Subgroup analysis of ECE-positive revealed that the diffuse pattern was associated with a lower total LN yield than the focal pattern (median 14 [IQR 11, 18] vs. median 18 [IQR 14, 24], P = 0.042), while no significant differences were observed in other nodal metrics (**Supplementary Table 2**).

Survival analysis revealed that patients with a nodal pathological response to neoadjuvant therapy exhibited comparable DFS and OS to those without a response. (all P > 0.05, **Supplementary Figure 8**). Further exploratory analysis within the ECE-positive subgroup demonstrated that survival outcomes did not differ significantly according to the number of ECE-positive LNs (all P > 0.05; **Supplementary Figure 9**). Similarly, the survival difference between focal and diffuse ECE patterns failed to reach statistical significance (all P > 0.05; **Supplementary Figure 10**).

### Cox regression analyses of factors associated with survival

To identify the independent prognostic factors associated with OS and DFS, univariate and multivariate Cox proportional hazards regression analyses were performed. Univariate analysis showed that histology, STAS, Visceral Pleural Invasion (VPI), Perineural Invasion (PNI), LVI, and ECE were significantly associated with DFS (all P < 0.05). For OS, PNI and LVI were identified as significant prognostic indicators. Variables for the multivariable models were selected based on their level of significance in the univariate analysis. Specifically, the top five most statistically significant variables were entered into the multivariable model for DFS, while the top two were included in the model for OS. In the multivariable Cox regression analysis for DFS, STAS (HR = 2.18, 95% CI: 1.05–4.53, P = 0.037), LVI (HR = 1.89, 95% CI: 1.03–3.48, P = 0.041), and ECE (HR = 2.37, 95% CI: 1.28–4.41, P = 0.006) were confirmed as reliable independent prognostic indicators (**Table 2**). Notably, although PNI achieved statistical significance (P < 0.001), it produced an anomalously inflated hazard ratio (HR = 19.14, 95% CI: 5.18–70.74), warranting cautious clinical interpretation. Regarding OS, the multivariable analysis confirmed LVI (HR = 3.99, 95% CI: 1.59–10.02, P = 0.003) as an independent predictor of poor survival. Consistent with the DFS model, although PNI again reached mathematical significance (P < 0.001), its anomalously inflated hazard ratio (HR = 13.22, 95% CI: 3.58–48.86), warranting cautious interpretation (**Table 3**). Furthermore, a sensitivity analysis incorporating all collected variables confirmed that ECE consistently remained an independent prognostic factor for DFS. This consistency across both the primary and the all-variable models reinforces the robustness of our primary conclusions (**Supplementary Tables 3**, **S4**).

**Table 2:** Cox proportional hazards regression model for DFS.

Characteristics	Univariate analysis		Multivariate analysis	
	CI	*P* value	CI	*P* value
Sex				
Female vs. Male	1.68 (0.71-3.96)	0.235		
Age				
≥65y vs. <65y	0.93 (0.53-1.62)	0.792		
Smoking History				
Yes vs. No	0.91 (0.51-1.63)	0.763		
ECOG				
1 vs. 0	1.07 (0.62-1.87)	0.805		
2 vs. 0	0 (0-Inf)	0.996		
Histology				
Squamous vs. Non-squamous	0.44 (0.25-0.77)	**0.004**	0.80 (0.39-1.63)	0.537
Surgical Type				
Non-lobectomy vs. Lobectomy	0.75 (0.40-1.38)	0.354		
Surgical Approach				
Open vs. MIS	0.60 (0.30-1.19)	0.144		
MPR vs. Non-MPR	0.60 (0.31-1.17)	0.136		
STAS vs. Non-STAS	3.20 (1.79-5.72)	**<0.001**	2.18 (1.05-4.53)	**0.037**
VPI vs. Non-VPI	2.30 (1.11-4.76)	**0.025**		
PNI vs. Non-PNI	7.86 (2.36-26.18)	**0.001**	19.14 (5.18-70.74)	**<0.001**
LVI vs. Non-LVI	2.81 (1.61-4.92)	**<0.001**	1.89 (1.03-3.48)	**0.041**
ypT				
ypT1 vs. ypT0	1.90 (0.56-6.48)	0.307		
ypT2 vs. ypT0	3.34 (1.00-11.12)	0.050		
ypT3 vs. ypT0	1.97 (0.47-8.25)	0.355		
ypT4 vs. ypT0	2.17 (0.34-13.72)	0.412		
ypN				
ypN2 vs. ypN1	1.38 (0.74-2.56)	0.315		
ypStage				
ypStage III vs. ypStage II	1.45 (0.74-2.84)	0.278		
Adjuvant chemotherapy				
Yes vs. No	0.92 (0.50-1.71)	0.799		
Adjuvant Immunotherapy				
Yes vs. No	1.04 (0.59-1.81)	0.900		
Extracapsular Extension				
Present vs. Absent	2.71 (1.54-4.76)	**0.001**	2.37 (1.28-4.41)	**0.006**

MPR, major pathologic response; STAS, Spread Through Air Spaces; VPI, Visceral Pleural Invasion; PNI, Perineural Invasion; LVI, Lymphovascular Invasion; ECOG, Eastern Cooperative Oncology Group Performance Status; MIS, Minimally Invasive Surgery. Bold values indicate statistically significant differences (*P* < 0.05).

**Table 3:** Cox proportional hazards regression model for OS.

Characteristics	Univariate analysis		Multivariate analysis	
	CI	*P* value	CI	*P* value
Sex				
Female vs. Male	1.16 (0.27-5.05)	0.841		
Age				
≥65y vs. <65y	0.93 (0.38-2.32)	0.881		
Smoking History				
Yes vs. No	1.95 (0.65-5.89)	0.237		
ECOG				
1 vs. 0	0.72 (0.29-1.77)	0.470		
2 vs. 0	0 (0-Inf)	0.998		
Histology				
Squamous vs. Non-squamous	0.63 (0.26-1.56)	0.322		
Surgical Type				
Non-lobectomy vs. Lobectomy	0.75 (0.27-2.09)	0.582		
Surgical Approach				
Open vs. MIS	0.96 (0.34-2.66)	0.934		
MPR vs. Non-MPR	0.46 (0.13-1.58)	0.216		
STAS vs. Non-STAS	1.13 (0.41-3.16)	0.810		
VPI vs. Non-VPI	2.18 (0.72-6.57)	0.167		
PNI vs. Non-PNI	13.87 (3.91-49.15)	**<0.001**	13.22 (3.58-48.86)	**<0.001**
LVI vs. Non-LVI	4.10 (1.64-10.22)	**0.002**	3.99 (1.59-10.02)	**0.003**
ypT				
ypT1 vs. ypT0	1.69 (0.20-14.50)	0.630		
ypT2 vs. ypT0	3.91 (0.50-30.57)	0.194		
ypT3 vs. ypT0	2.59 (0.23-28.63)	0.437		
ypT4 vs. ypT0	3.82 (0.24-61.39)	0.344		
ypN				
ypN2 vs. ypN1	1.46 (0.52-4.05)	0.471		
ypStage				
ypStage III vs. ypStage II	2.12 (0.62-7.29)	0.232		
Adjuvant chemotherapy				
Yes vs. No	0.70 (0.26-1.85)	0.469		
Adjuvant Immunotherapy				
Yes vs. No	0.68 (0.27-1.66)	0.394		
Extracapsular Extension				
Present vs. Absent	1.65 (0.67-4.07)	0.275		

MPR, major pathologic response; STAS, Spread Through Air Spaces; VPI, Visceral Pleural Invasion; PNI, Perineural Invasion; LVI, Lymphovascular Invasion; ECOG, Eastern Cooperative Oncology Group Performance Status; MIS, Minimally Invasive Surgery. Bold values indicate statistically significant differences (*P* < 0.05).

### Prognostic value of ECE across different neoadjuvant treatment subgroups

A total of seven ICIs were administered in our cohort, with their distribution summarized in **Supplementary Figure 11**. As the durvalumab subgroup included only three patients, it was excluded from the subgroup analysis. When stratified according to the remaining six individual ICIs, ECE-positive patients showed a trend toward inferior DFS across different ICI treatment subgroups. This unfavorable DFS trend reached statistical significance in the tislelizumab and toripalimab subgroups, whereas the other four ICI subgroups demonstrated similar tendencies toward poorer DFS among ECE-positive patients without reaching statistical significance. No significant differences in OS were observed across any of the six ICI subgroups (**Supplementary Figures 12**, **S13**).

Furthermore, when patients were stratified according to the number of completed neoadjuvant treatment cycles, ECE remained significantly associated with inferior DFS among patients receiving ≥3 cycles of neoadjuvant therapy (P = 0.002), while no significant difference in OS was observed (P = 0.522). Similarly, among patients receiving <3 cycles of neoadjuvant therapy, ECE-positive patients exhibited a trend toward shorter DFS, although statistical significance was not reached (P = 0.053), and no significant difference in OS was observed (P = 0.355) (**Supplementary Figure 14**).

### Evaluation of the combined ECE-MPR predictive model for DFS

**Figure 6:**
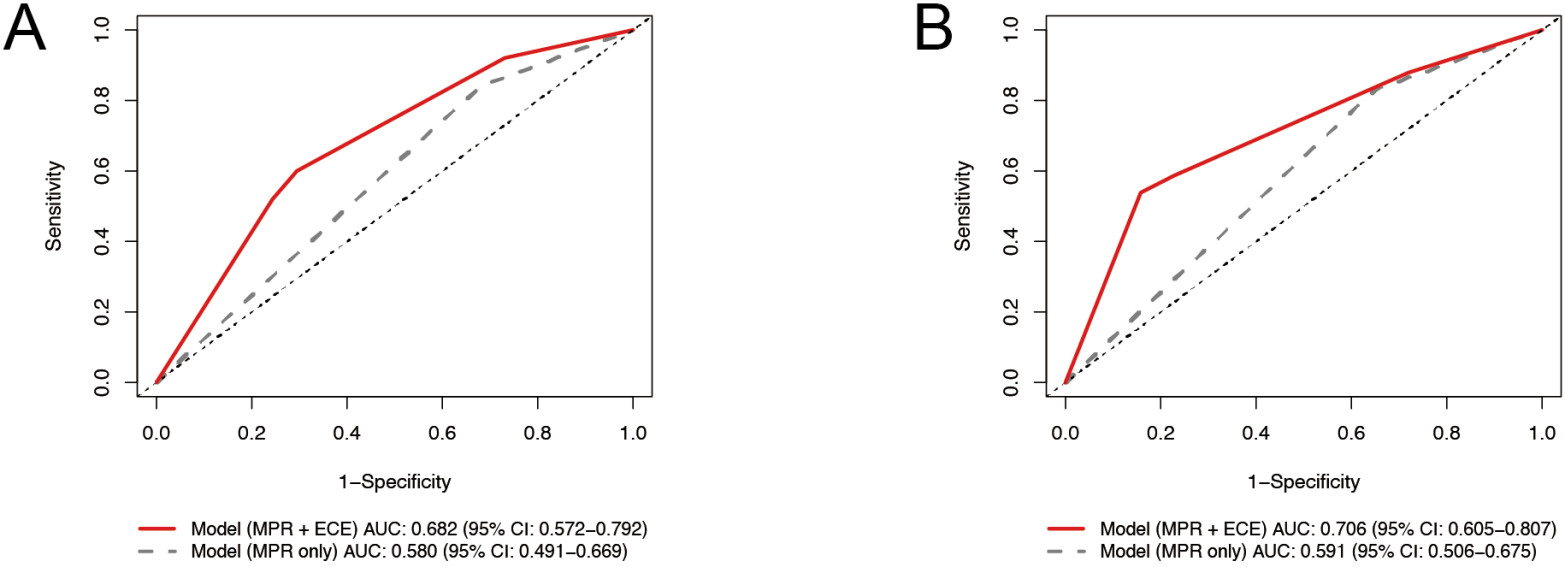
Time-dependent ROC curves predicting DFS. **(A)** 1-year and **(B)** 2-year DFS prediction models.

To further elucidate whether combining ECE and neoadjuvant pathological response could yield enhanced prognostic precision, we established predictive models and executed time-dependent ROC analyses for DFS. Intriguingly, the combined prediction model incorporating both MPR and ECE status exhibited substantially augmented discriminatory capacity compared to the model utilizing MPR alone. For 1-year DFS prediction, the combined model achieved an AUC of 0.682 (95% CI: 0.572–0.792), contrasting with an AUC of 0.580 (95% CI: 0.491–0.669) for the MPR-only model (**Figure 6A**). This synergistic effect was even more pronounced in predicting 2-year DFS, where the addition of ECE elevated the AUC from 0.591 (95% CI: 0.506–0.675) to 0.706 (95% CI: 0.605–0.807) (**Figure 6B**). These findings firmly demonstrate that incorporating post-surgical nodal ECE status serves as an effective adjunct to pathological response, remarkably refining the post-neoadjuvant risk stratification for non-small cell lung cancer.

### Prognostic analysis after PSM

To mitigate the potential confounding effects of baseline imbalances between the groups, a PSM analysis was performed. After matching, a total of 60 patients were included in the adjusted cohort, comprising 34 patients in the ECE-negative group and 26 patients in the ECE-positive group. The balance of covariates between the two groups was thoroughly evaluated and significantly improved, as illustrated by the Love plot (**Supplementary Figure 15**). Furthermore, as detailed in **Supplementary Table 5**, no statistically significant differences remained across any of the clinical or pathological characteristics (all P > 0.05). In the overall propensity score-matched cohort, patients with ECE still exhibited significantly inferior DFS compared to those without ECE (P = 0.004). However, no significant difference was observed in OS between the two groups (P = 0.110) (**Supplementary Figure 16**). In the N1 subgroup, the previously observed significant difference in DFS became non-significant (P = 0.119), while OS remained statistically comparable (P = 0.822). Within the N2 subgroup, ECE positivity was significantly associated with both poorer OS (P = 0.042) and DFS (P = 0.011) (**Supplementary Figure 17**). Interestingly, in the squamous cell carcinoma subgroup, OS transitioned from non-significant to significantly worse for ECE-positive patients (P = 0.035), whereas the difference in DFS became marginally non-significant (P = 0.051). Meanwhile, the prognostic trends in the adenocarcinoma subgroup remained consistent with the pre-matched analysis (**Supplementary Figure 18**).

To mitigate the impact of potential selection bias in evaluating the efficacy of adjuvant therapies, we conducted an additional PSM analysis based on the receipt of adjuvant immunotherapy or chemotherapy. The covariate balance between groups was comprehensively assessed and significantly improved, as illustrated by the Love plot (**Supplementary Figure 19**). Consistent with the pre-match findings, the PSM-adjusted analyses confirmed that neither adjuvant immunotherapy nor chemotherapy conferred a statistically significant therapeutic benefit in either ECE-positive or ECE-negative patients (all P > 0.05) (**Supplementary Figures 20**, **S21**).

## Discussion

This study evaluated the prognostic impact of ECE in NSCLC patients following NCIT. Patients with ECE exhibited significantly shorter DFS, a finding consistent across various histological subtypes and nodal stages. Multivariate analysis confirmed ECE as an independent predictor of recurrence, associated with a more than two-fold increased risk. Notably, ECE status did not significantly influence OS. Instead, the multivariate analysis established PNI and LVI as independent prognostic predictors for OS.

In recent years, accumulating evidence has demonstrated that ECE is significantly associated with an increased risk of recurrence in NSCLC. A study involving 282 patients with stage III-N2 NSCLC demonstrated that, after balancing baseline characteristics using PSM, ECE remained an independent risk factor for inferior DFS (HR = 1.629) (15). In another large cohort of 1,713 patients who underwent lobectomy or pneumonectomy for NSCLC, the authors reported that the prevalence of ECE increased with advancing pN category and that the presence of ECE was significantly associated with an increased risk of RFS events (16). Furthermore, a meta-analysis conducted by Luchini et al., including 13 studies and 1,709 patients, demonstrated that ECE was significantly associated with a higher risk of disease recurrence, suggesting that tumor extension beyond the lymph node capsule reflects a more aggressive tumor biological phenotype (17). However, none of these studies included patients receiving NCIT. Given that NCIT can substantially reshape the tumor immune microenvironment, whether ECE retains its prognostic significance after NCIT remains uncertain (18). Our study demonstrated that even after receiving NCIT, patients with ECE continued to exhibit a significantly higher risk of recurrence compared with those without ECE, and ECE remained an independent prognostic factor for DFS. These findings extend the prognostic significance of ECE to the NCIT setting, indicating that despite the administration of preoperative systemic therapy, ECE remains a high-risk pathological feature associated with tumor recurrence.

However, in our cohort, the impact of ECE on OS did not reach statistical significance, which differs from previous studies. For instance, a large-scale cohort of 4,061 patients with NSCLC demonstrated that ECE correlates with both diminished OS and DFS (13). Similarly, Lee and colleagues reported five-year survival rates of 40.7% for ECE-negative patients compared to only 13.5% for ECE-positive individuals (19). A study including 388 patients with stage IIA–IIIA NSCLC demonstrated that ECE was an independent prognostic factor and was associated with significantly shorter median OS (30 vs. 43 months) (20). In addition, Chen et al. investigated 279 patients with lung adenocarcinoma and reported that patients with ECE had significantly worse OS compared with those without ECE, with ECE identified as an independent predictor of OS (21). Furthermore, a large-scale cohort study also confirmed that ECE was an independent risk factor for inferior OS among all patients with pathological lymph node-positive disease after adjustment for relevant covariates (16). Several factors unique to the NCIT era likely contribute to this discrepancy. First, NCIT reshapes the tumor microenvironment, potentially rendering ECE-associated residual disease more sensitive to subsequent treatments upon relapse, thereby preserving OS (4, 22). Second, this phenomenon likely reflects the rapid therapeutic evolution in recent years. Unlike historical cohorts, contemporary patients benefit from sophisticated post-recurrence regimens, including antibody-drug conjugates (ADCs) and next-generation targeted therapies. These modern interventions significantly prolong post-progression survival, effectively mitigating the survival disadvantage typically triggered by ECE-related recurrence (23, 24). Consequently, within our study cohort, ECE appears to function primarily as a predictor of early recurrence rather than a definitive driver of overall mortality in the NCIT era.

Our results demonstrated that patients with ECE had a significantly lower MPR rate following NCIT compared with those without ECE (15.4% vs. 35.4%, P = 0.028). This finding may be explained by the distinct tumor microenvironmental characteristics associated with ECE, as reported in previous pathological studies. Several studies have shown that ECE-positive tumors are characterized by reduced infiltration of tumor-infiltrating lymphocytes (TILs), suggesting impaired local anti-tumor immune surveillance. Noda et al. reported that low TIL infiltration was significantly associated with the presence of ECE, and the immune profiles of primary tumors were highly concordant with those observed in metastatic lymph nodes and ECE lesions (25). Similarly, Bulic et al. demonstrated that patients with ECE exhibited significantly lower stromal TIL levels at the invasive margin compared with ECE-negative patients (26). As a key component of chemoimmunotherapy, the efficacy of immune checkpoint blockade largely depends on the presence and functional activity of endogenous tumor-infiltrating lymphocytes. Tumors with limited lymphocyte infiltration are more likely to develop an immunosuppressive microenvironment, which may contribute to reduced sensitivity to neoadjuvant chemoimmunotherapy (27). This may mechanically explain the substantially lower MPR rate observed in patients with ECE.

Frequent local recurrence despite conventional R0 status led the IASLC to propose reclassifying ECE from an R0 category to an incomplete or uncertain category. While previous large-scale studies confirmed that ECE-positive individuals carry recurrence comparable to those of R1 resection, those analyses excluded patients receiving neoadjuvant therapy (13). Notably, our findings extend this evidence to the NCIT setting, demonstrating that ECE-positive patients exhibit significantly inferior DFS compared to their ECE-negative R0 counterparts. These observations provide preliminary clinical evidence supporting the proposed reclassification within the context of NCIT. Nevertheless, given the modest sample size and the absence of a statistically significant OS difference, definitive conclusions warrant further validation through larger, prospective trials.

In our multivariate analysis, PNI demonstrated statistical significance for both DFS and OS; however, we caution against definitively interpreting it as an independent prognostic factor due to anomalously inflated HRs. This phenomenon is primarily attributed to the fact that all three PNI-positive patients in our cohort experienced both disease recurrence and mortality. Although this 100% event rate suggests that PNI may serve as a surrogate for extreme biological aggressiveness in the post-NCIT setting, such absolute correlation within a small subgroup leads to quasi-complete separation in the Cox model. This inherently inflates the risk estimates and substantially widens the confidence intervals. Consequently, we interpret PNI as a potent prognostic indicator that warrants further validation in larger cohorts.

The study had several limitations. First, its retrospective and single-center design may introduce selection bias and limit the generalizability of the findings. Second, heterogeneity in neoadjuvant and adjuvant treatment regimens could introduce confounding factors. Third, our subgroup analyses were underpowered due to the limited number of cases. Furthermore, the events-per-variable (EPV) ratio in our multivariable analysis was relatively low. This limited number of events, combined with the 100% event concordance observed for rare features such as PNI, may have resulted in instability of the Cox regression model and consequently led to inflated HR estimates.

Our findings support the routine integration of ECE status into pathological assessment and post-NCIT risk stratification for NSCLC. Heightened vigilance is required regarding the elevated risk of recurrence in ECE-positive individuals. Furthermore, the identification of LVI as an independent predictor of OS underscores the necessity for rigorous post-operative follow-up and personalized management to mitigate mortality risk and enhance overall clinical outcomes.

## Conclusion

ECE significantly impairs DFS and serves as an independent prognostic factor for DFS in patients receiving NCIT. Additionally, LVI is an independent prognostic factor for OS. Our findings suggest that intensified postoperative surveillance for recurrence is warranted for ECE-positive patients.

## Data Availability

The raw data supporting the conclusions of this article will be made available by the authors, without undue reservation.

## Supplementary material

Supplementary Figure 1 Interobserver agreement for pathological assessment of ECE. Agreement between two independent pathologists was evaluated using Cohen’s kappa coefficient.

Supplementary Figure 2 Adjuvant immunotherapy impact by ECE status. **(A, B)** ECE positive; **(C, D)** ECE negative. Panels **(A, C)** show OS; **(B, D)** show DFS.

Supplementary Figure 3 Adjuvant immunotherapy in squamous cell carcinoma by ECE status. **(A, B)** ECE positive; **(C, D)** ECE negative. Panels **(A, C)** show OS; **(B, D)** show DFS.

Supplementary Figure 4 Adjuvant immunotherapy in adenocarcinoma by ECE status. **(A, B)** ECE positive; **(C, D)** ECE negative. Panels **(A, C)** show OS; **(B, D)** show DFS.

Supplementary Figure 5 Adjuvant chemotherapy impact by ECE status. **(A, B)** ECE positive; **(C, D)** ECE negative. Panels **(A, C)** show OS; **(B, D)** show DFS.

Supplementary Figure 6 Adjuvant chemotherapy in squamous cell carcinoma by ECE status. **(A, B)** ECE positive; **(C, D)** ECE negative. Panels **(A, C)** show OS; **(B, D)** show DFS.

Supplementary Figure 7 Adjuvant chemotherapy in adenocarcinoma by ECE status. (A, B) ECE positive; (C, D) ECE negative. Panels **(A, C)** show OS; **(B, D)** show DFS.

Supplementary Figure 8 Survival outcomes stratified by nodal pathological response to nodal pathological response following neoadjuvant chemoimmunotherapy. **(A)** OS; **(B)** DFS.

Supplementary Figure 9 Survival outcomes stratified by numbers of ECE-Positive LNs. **(A)** OS; **(B)** DFS.

Supplementary Figure 10 Survival outcomes stratified by morphological patterns of ECE. **(A)** OS; **(B)** DFS.

Supplementary Figure 11 Distribution of specific immune checkpoint inhibitors (ICIs) utilized for neoadjuvant therapy.

Supplementary Figure 12 Survival outcomes by ECE status in different ICI subgroups **(A, B)** Camrelizumab; **(C, D)** Nivolumab; **(E, F)** Pembrolizumab. Panels **(A, C, E)** show OS; **(B, D, F)** show DFS.

Supplementary Figure 13 Survival outcomes by ECE status in different ICI subgroups **(A, B)** Sintilimab; **(C, D)** Tislelizumab; **(E, F)** Toripalimab. Panels **(A, C, E)** show OS; **(B, D, F)** show DFS.

Supplementary Figure 14 Survival outcomes by ECE status according to the number of completed neoadjuvant therapy cycles **(A, B)** <3 cycles; **(C, D)** ≥3 cycles. Panels **(A, C)** show OS; **(B, D)** show DFS.

Supplementary Figure 15 Love plot visualizing covariate balance before and after 1:2 propensity score matching (PSM) based on ECE status.

Supplementary Figure 16 Survival outcomes by ECE status in all patients after PSM. **(A)** OS; **(B)** DFS.

Supplementary Figure 17 Survival by ECE status and N stage after PSM. **(A, B)** N1 stage; **(C, D)** N2 stage. Panels **(A, C)** show OS; **(B, D)** show DFS.

Supplementary Figure 18 Survival by ECE status and histology after PSM. **(A, B)** Squamous cell carcinoma; **(C, D)** Adenocarcinoma. Panels **(A, C)** show OS; **(B, D)** show DFS.

Supplementary Figure 19 Love plot visualizing covariate balance before and after 1:1 PSM based on adjuvant therapy. **(A)** Adjuvant immunotherapy; **(B)** Adjuvant chemotherapy.

Supplementary Figure 20 Adjuvant immunotherapy impact by ECE status after PSM. **(A, B)** ECE positive; **(C, D)** ECE negative. Panels **(A, C)** show OS; **(B, D)** show DFS.

Supplementary Figure 21 Adjuvant chemotherapy impact by ECE status after PSM. **(A, B)** ECE positive; **(C, D)** ECE negative. Panels **(A, C)** show OS; **(B, D)** show DFS.
