# Supplementary material for: Prognostic significance of extracapsular extension in patients with non-small cell lung cancer following neoadjuvant chemoimmunotherapy: a retrospective cohort study
Source: Front Immunol. 2026 Jul 15;17:1799856. doi: 10.3389/fimmu.2026.1799856 (PMC13415941; doi:10.3389/fimmu.2026.1799856)
Supplement: Supplementary Figure 1 — Interobserver agreement for pathological assessment of ECE. Agreement between two independent pathologists was evaluated using Cohen’s kappa coefficient. [file SupplementaryFile1.zip › Supplementary Tables.DOCX]

**Table S1** Comparison of nodal burden metrics between ECE-negative and ECE-positive patients.

| **Characteristic** | **Overall** N = 104 | **ECE Negative** N = 65 | **ECE Positive** N = 39 | ***p* Value** |
| --- | --- | --- | --- | --- |
| **Total LNs resected** | 18 (13, 22) | 18 (13, 22) | 18 (13, 22) | 0.793 |
| **Metastatic LNs count** | 2 (1, 5) | 2 (1, 4) | 3 (2, 6) | **0.006** |
| **ECE+ LNs count** | 0 (0, 1) | — | 1 (1, 2) | — |

ECE: Extracapsular Extension; LN: Lymph nodes.

**Table S2** Comparison of nodal burden metrics between focal and diffuse ECE morphological patterns.

| **Characteristic** | **ECE Positive** N = 39 | **Region** N = 31 | **Diffuse**  N = 8 | ***p* Value** |
| --- | --- | --- | --- | --- |
| **Total LNs resected** | 18 (13, 22) | 18 (14, 24) | 14 (11, 18) | **0.042** |
| **Metastatic LNs count** | 3 (2, 6) | 3 (2, 6) | 4 (1, 6) | 0.659 |
| **ECE+ LNs count** | 1 (1, 2) | 1 (1, 2) | 1 (1, 2) | 0.493 |

ECE: Extracapsular Extension; LN: Lymph nodes.

**Table S3** Cox Proportional Hazards Regression Model for DFS

| Characteristics | Univariate Analysis | | Multivariate Analysis | |
| --- | --- | --- | --- | --- |
|  | CI | *p* Value | CI | *p* Value |
| Sex |  |  |  |  |
| Female vs. Male | 1.68 (0.71-3.96) | 0.235 | 1.15 (0.37-3.52) | 0.813 |
| Age |  |  |  |  |
| ≥65y vs. <65y | 0.93 (0.53-1.62) | 0.792 | 1.01 (0.97-1.05) | 0.722 |
| Smoking History |  |  |  |  |
| Yes vs. No | 0.91 (0.51-1.63) | 0.763 | 2.09 (0.84-5.19) | 0.114 |
| ECOG |  |  |  |  |
| 1 vs. 0 | 1.07 (0.62-1.87) | 0.805 | 1.49 (0.75-2.97) | 0.253 |
| 2 vs. 0 | 0 (0-Inf) | 0.996 | 0 (0-Inf) | 0.996 |
| Histology |  |  |  |  |
| Squamous vs. Non-squamous | 0.44 (0.25-0.77) | **0.004** | 0.51 (0.21-1.26) | 0.147 |
| Surgical Type |  |  |  |  |
| Non-lobectomy vs. Lobectomy | 0.75 (0.40-1.38) | 0.354 | 1.20 (0.46-3.17) | 0.708 |
| Surgical Approach |  |  |  |  |
| Open vs. MIS | 0.60 (0.30-1.19) | 0.144 | 0.89 (0.35-2.24) | 0.803 |
| MPR vs. Non-MPR | 0.60 (0.31-1.17) | 0.136 | 2.08 (0.73-5.91) | 0.170 |
| STAS vs. Non-STAS | 3.20 (1.79-5.72) | **<0.001** | 2.46 (0.99-6.15) | 0.053 |
| VPI vs. Non-VPI | 2.30 (1.11-4.76) | **0.025** | 1.45 (0.54-3.94) | 0.461 |
| PNI vs. Non-PNI | 7.86 (2.36-26.18) | **0.001** | 36.75 (6.83-197.70) | **<0.001** |
| LVI vs. Non-LVI | 2.81 (1.61-4.92) | **<0.001** | 2.72 (1.23-5.97) | **0.013** |
| ypT |  |  |  |  |
| ypT1 vs. ypT0 | 1.90 (0.56-6.48) | 0.307 | 1.54 (0.35-6.79) | 0.567 |
| ypT2 vs. ypT0 | 3.34 (1.00-11.12) | 0.050 | 2.76 (0.62-12.36) | 0.185 |
| ypT3 vs. ypT0 | 1.97 (0.47-8.25) | 0.355 | 0.72 (0.08-6.64) | 0.774 |
| ypT4 vs. ypT0 | 2.17 (0.34-13.72) | 0.412 | 1.47 (0.15-14.80) | 0.745 |
| ypN |  |  |  |  |
| ypN2 vs. ypN1 | 1.38 (0.74-2.56) | 0.315 | 0.61 (0.08-4.85) | 0.640 |
| ypStage |  |  |  |  |
| ypStage III vs. ypStage II | 1.45 (0.74-2.84) | 0.278 | 1.20 (0.13-11.13) | 0.870 |
| Adjuvant chemotherapy |  |  |  |  |
| Yes vs. No | 0.92 (0.50-1.71) | 0.799 | 0.52 (0.22-1.25) | 0.146 |
| Adjuvant Immunotherapy |  |  |  |  |
| Yes vs. No | 1.04 (0.59-1.81) | 0.900 | 1.66 (0.70-3.92) | 0.249 |
| Extracapsular Extension |  |  |  |  |
| Present vs. Absent | 2.71 (1.54-4.76) | **0.001** | 2.58 (1.33-5.01) | **0.005** |

MPR: major pathologic response; STAS: Spread Through Air Spaces; VPI: Visceral Pleural Invasion; PNI: Perineural Invasion; LVI: Lymphovascular Invasion; ECOG: **Eastern Cooperative Oncology Group Performance Status**; MIS: Minimally Invasive Surgery.

**Table S4** Cox Proportional Hazards Regression Model for OS

| Characteristics | Univariate Analysis | | Multivariate Analysis | |
| --- | --- | --- | --- | --- |
|  | CI | *p* Value | CI | *p* Value |
| Sex |  |  |  |  |
| Female vs. Male | 1.16 (0.27-5.05) | 0.841 | 0.52 (0.05-5.15) | 0.576 |
| Age |  |  |  |  |
| ≥65y vs. <65y | 0.93 (0.38-2.32) | 0.881 | 1.01 (0.94-1.08) | 0.790 |
| Smoking History |  |  |  |  |
| Yes vs. No | 1.95 (0.65-5.89) | 0.237 | 4.96 (0.69-35.84) | 0.112 |
| ECOG |  |  |  |  |
| 1 vs. 0 | 0.72 (0.29-1.77) | 0.470 | 2.08 (0.50-8.67) | 0.313 |
| 2 vs. 0 | 0 (0-Inf) | 0.998 | 0 (0-Inf) | 0.998 |
| Histology |  |  |  |  |
| Squamous vs. Non-squamous | 0.63 (0.26-1.56) | 0.322 | 0.43 (0.10-1.91) | 0.270 |
| Surgical Type |  |  |  |  |
| Non-lobectomy vs. Lobectomy | 0.75 (0.27-2.09) | 0.582 | 0.53 (0.08-3.37) | 0.504 |
| Surgical Approach |  |  |  |  |
| Open vs. MIS | 0.96 (0.34-2.66) | 0.934 | 1.91 (0.34-10.75) | 0.462 |
| MPR vs. Non-MPR | 0.46 (0.13-1.58) | 0.216 | 1.48 (0.22-10.05) | 0.687 |
| STAS vs. Non-STAS | 1.13 (0.41-3.16) | 0.810 | 1.19 (0.18-7.96) | 0.861 |
| VPI vs. Non-VPI | 2.18 (0.72-6.57) | 0.167 | 1.30 (0.25-6.80) | 0.757 |
| PNI vs. Non-PNI | 13.87 (3.91-49.15) | **<0.001** | 158.44 (9.76-2571.93) | **<0.001** |
| LVI vs. Non-LVI | 4.10 (1.64-10.22) | **0.002** | 6.36 (1.53-26.47) | **0.011** |
| ypT |  |  |  |  |
| ypT1 vs. ypT0 | 1.69 (0.20-14.50) | 0.630 | 1.23 (0.07-20.34) | 0.885 |
| ypT2 vs. ypT0 | 3.91 (0.50-30.57) | 0.194 | 5.48 (0.31-98.28) | 0.248 |
| ypT3 vs. ypT0 | 2.59 (0.23-28.63) | 0.437 | 0 (0-Inf) | 0.998 |
| ypT4 vs. ypT0 | 3.82 (0.24-61.39) | 0.344 | 1.39 (0.02-84.55) | 0.875 |
| ypN |  |  |  |  |
| ypN2 vs. ypN1 | 1.46 (0.52-4.05) | 0.471 | 0 (0-Inf) | 0.998 |
| ypStage |  |  |  |  |
| ypStage III vs. ypStage II | 2.12 (0.62-7.29) | 0.232 | 11327114.79 (0-Inf) | 0.998 |
| Adjuvant chemotherapy |  |  |  |  |
| Yes vs. No | 0.70 (0.26-1.85) | 0.469 | 0.42 (0.09-1.88) | 0.257 |
| Adjuvant Immunotherapy |  |  |  |  |
| Yes vs. No | 0.68 (0.27-1.66) | 0.394 | 2.44 (0.46-13.07) | 0.297 |
| Extracapsular Extension |  |  |  |  |
| Present vs. Absent | 1.65 (0.67-4.07) | 0.275 | 1.36 (0.44-4.17) | 0.593 |

MPR: major pathologic response; STAS: Spread Through Air Spaces; VPI: Visceral Pleural Invasion; PNI: Perineural Invasion; LVI: Lymphovascular Invasion; ECOG: **Eastern Cooperative Oncology Group Performance Status**; MIS: Minimally Invasive Surgery.

**Table S5** Characteristics of Patients With NSCLC Stratified by ECE after PSM

| **Characteristic** | **Overall**  N = 60 | **ECE Negative**  N = 34 | **ECE Positive**  N = 26 | ***p* Value** |
| --- | --- | --- | --- | --- |
| Age | 63.0 (56.0, 67.0) | 63.0 (56.0, 67.0) | 63.5 (56.0, 67.0) | >0.999 |
| Sex |  |  |  | >0.999 |
| male | 56 (93.3%) | 32 (94.1%) | 24 (92.3%) |  |
| female | 4 (6.7%) | 2 (5.9%) | 2 (7.7%) |  |
| Smoking |  |  |  | 0.909 |
| No | 18 (30.0%) | 10 (29.4%) | 8 (30.8%) |  |
| Yes | 42 (70.0%) | 24 (70.6%) | 18 (69.2%) |  |
| ECOG |  |  |  | 0.505 |
| 0 | 26 (43.3%) | 16 (47.1%) | 10 (38.5%) |  |
| 1 | 34 (56.7%) | 18 (52.9%) | 16 (61.5%) |  |
| Histology |  |  |  | 0.750 |
| Adenocarcinoma | 24 (40.0%) | 13 (38.2%) | 11 (42.3%) |  |
| Squamous cell carcinoma | 36 (60.0%) | 21 (61.8%) | 15 (57.7%) |  |
| Surgical approach |  |  |  | 0.265 |
| Open | 15 (25.0%) | 9 (26.5%) | 6 (23.1%) |  |
| VATS | 40 (66.7%) | 24 (70.6%) | 16 (61.5%) |  |
| RATS | 5 (8.3%) | 1 (2.9%) | 4 (15.4%) |  |
| Surgical type |  |  |  | 0.450 |
| Lobectomy | 43 (71.7%) | 23 (67.6%) | 20 (76.9%) |  |
| Sleeve lobectomy | 11 (18.3%) | 6 (17.6%) | 5 (19.2%) |  |
| Pneumonectomy | 6 (10.0%) | 5 (14.7%) | 1 (3.8%) |  |
| MPR |  |  |  | 0.689 |
| No | 47 (78.3%) | 26 (76.5%) | 21 (80.8%) |  |
| Yes | 13 (21.7%) | 8 (23.5%) | 5 (19.2%) |  |
| STAS |  |  |  | 0.495 |
| No | 42 (70.0%) | 25 (73.5%) | 17 (65.4%) |  |
| Yes | 18 (30.0%) | 9 (26.5%) | 9 (34.6%) |  |
| VPI |  |  |  | >0.999 |
| No | 56 (93.3%) | 32 (94.1%) | 24 (92.3%) |  |
| Yes | 4 (6.7%) | 2 (5.9%) | 2 (7.7%) |  |
| PNI |  |  |  | / |
| No | 60 (100.0%) | 34 (100.0%) | 26 (100.0%) |  |
| LVI |  |  |  | 0.714 |
| No | 43 (71.7%) | 25 (73.5%) | 18 (69.2%) |  |
| Yes | 17 (28.3%) | 9 (26.5%) | 8 (30.8%) |  |
| ypT |  |  |  | 0.822 |
| 0 | 7 (11.7%) | 4 (11.8%) | 3 (11.5%) |  |
| 1 | 21 (35.0%) | 14 (41.2%) | 7 (26.9%) |  |
| 2 | 26 (43.3%) | 13 (38.2%) | 13 (50.0%) |  |
| 3 | 4 (6.7%) | 2 (5.9%) | 2 (7.7%) |  |
| 4 | 2 (3.3%) | 1 (2.9%) | 1 (3.8%) |  |
| ypN |  |  |  | 0.986 |
| 1 | 23 (38.3%) | 13 (38.2%) | 10 (38.5%) |  |
| 2 | 37 (61.7%) | 21 (61.8%) | 16 (61.5%) |  |
| ypStage |  |  |  | 0.896 |
| II | 19 (31.7%) | 11 (32.4%) | 8 (30.8%) |  |
| III | 41 (68.3%) | 23 (67.6%) | 18 (69.2%) |  |
| Adjuvant chemotherapy |  |  |  | 0.689 |
| No | 13 (21.7%) | 8 (23.5%) | 5 (19.2%) |  |
| Yes | 47 (78.3%) | 26 (76.5%) | 21 (80.8%) |  |
| Adjuvant immunotherapy |  |  |  | 0.580 |
| No | 23 (38.3%) | 12 (35.3%) | 11 (42.3%) |  |
| Yes | 37 (61.7%) | 22 (64.7%) | 15 (57.7%) |  |

ECE: Extracapsular Extension; MPR: major pathologic response; STAS: Spread Through Air Spaces; VPI: Visceral Pleural Invasion; PNI: Perineural Invasion; LVI: Lymphovascular Invasion; ECOG: **Eastern Cooperative Oncology Group Performance Status**; RATS: robot-assisted thoracic surgery; VATS: video-assisted thoracoscopic surgery.
