# Supplementary figures and images for: Prognostic significance of extracapsular extension in patients with non-small cell lung cancer following neoadjuvant chemoimmunotherapy: a retrospective cohort study
Source: Front Immunol. 2026 Jul 15;17:1799856. doi: 10.3389/fimmu.2026.1799856 (PMC13415941; doi:10.3389/fimmu.2026.1799856)

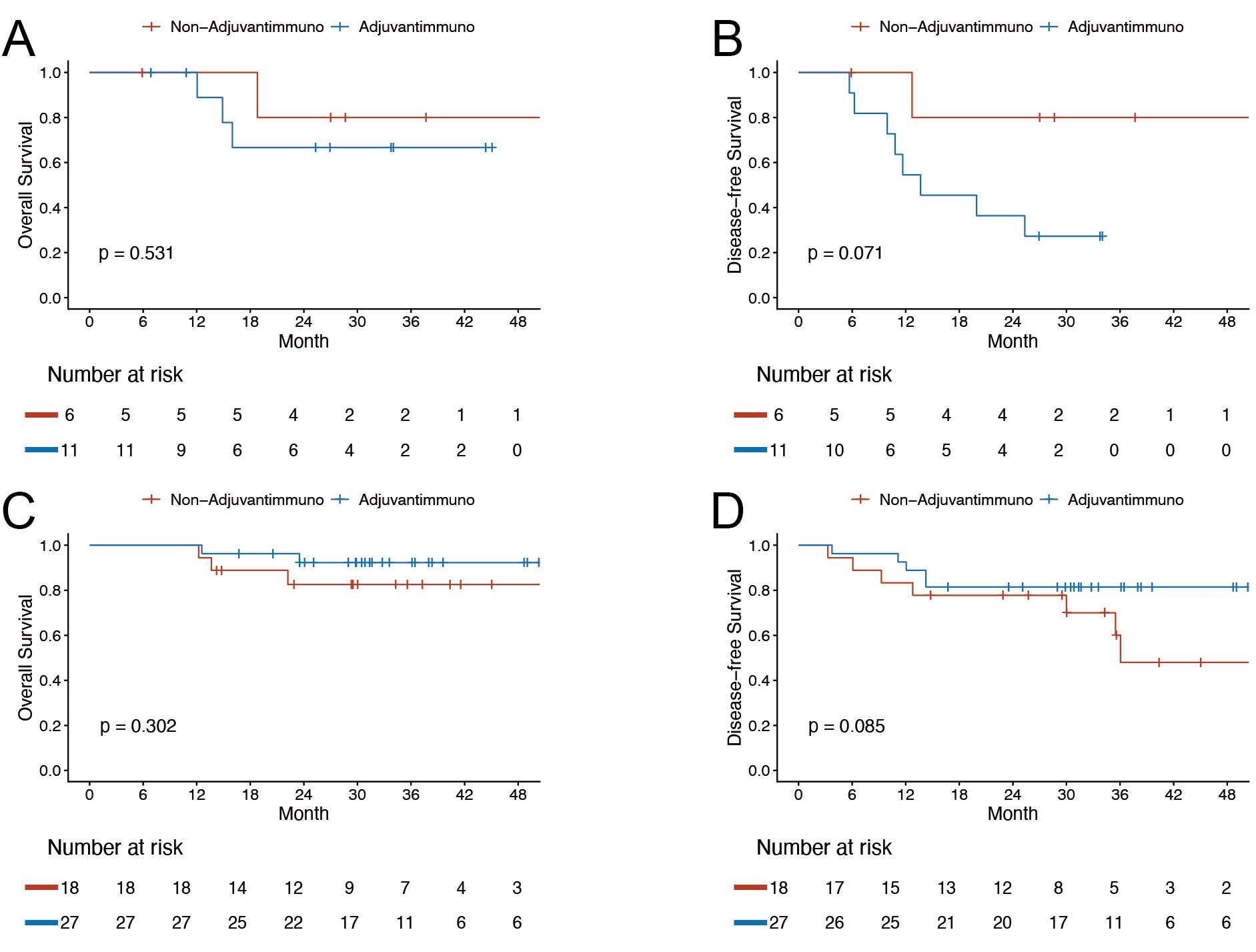

Supplement: Supplementary Figure 1 — Interobserver agreement for pathological assessment of ECE. Agreement between two independent pathologists was evaluated using Cohen’s kappa coefficient. [file SupplementaryFile1.zip › Supplementary Figure 3.PNG]

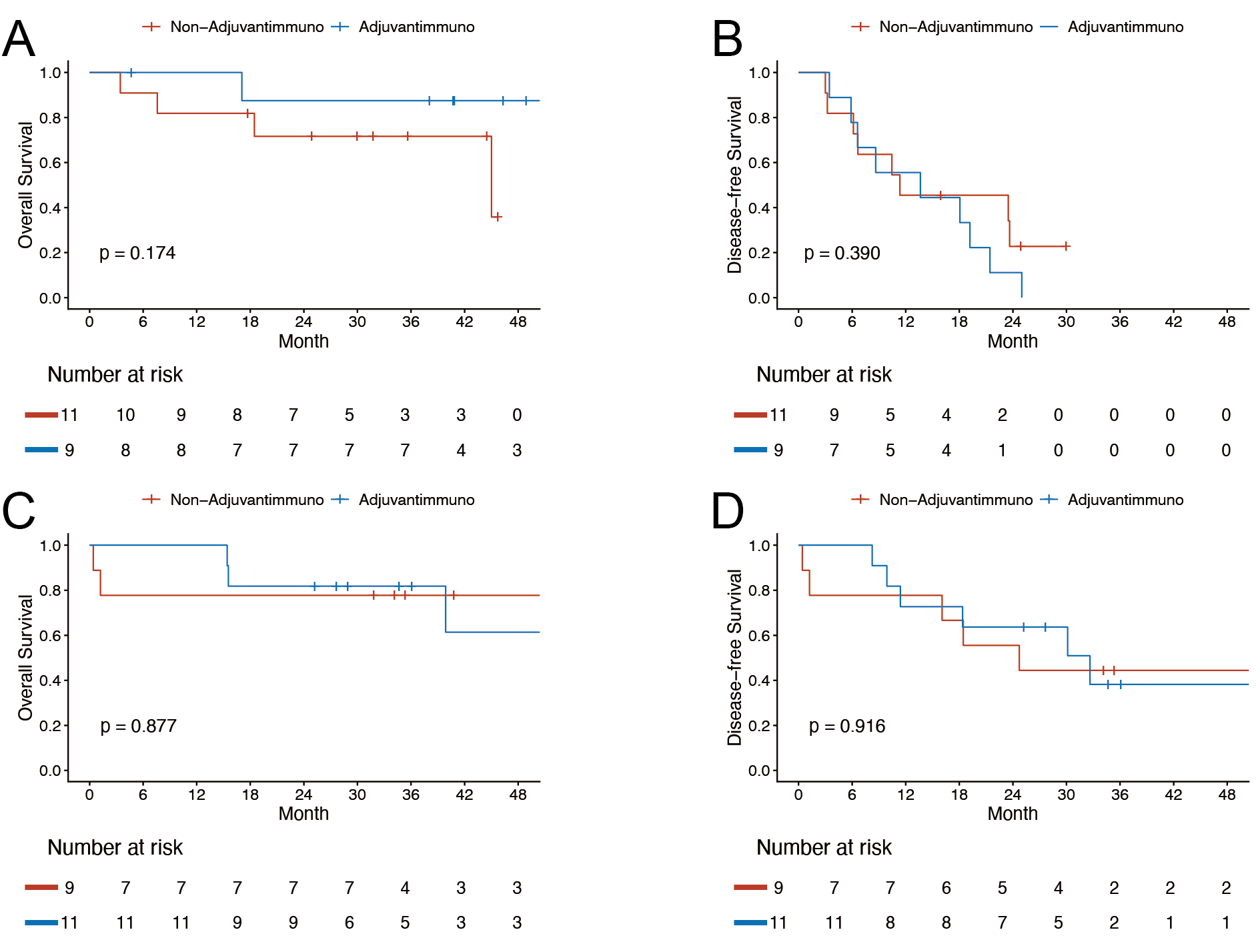

Supplement: Supplementary Figure 1 — Interobserver agreement for pathological assessment of ECE. Agreement between two independent pathologists was evaluated using Cohen’s kappa coefficient. [file SupplementaryFile1.zip › Supplementary Figure 4.PNG]

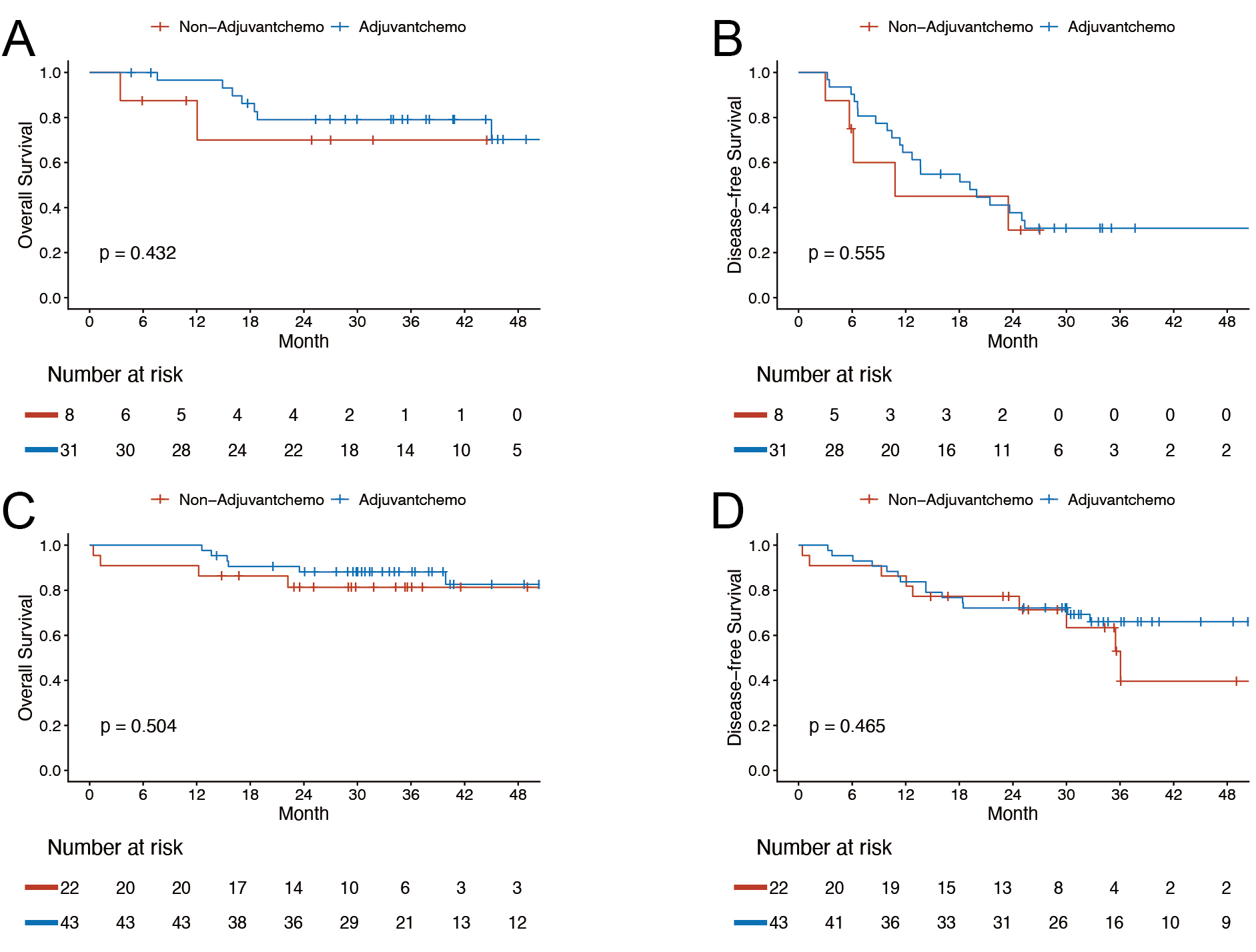

Supplement: Supplementary Figure 1 — Interobserver agreement for pathological assessment of ECE. Agreement between two independent pathologists was evaluated using Cohen’s kappa coefficient. [file SupplementaryFile1.zip › Supplementary Figure 5.PNG]

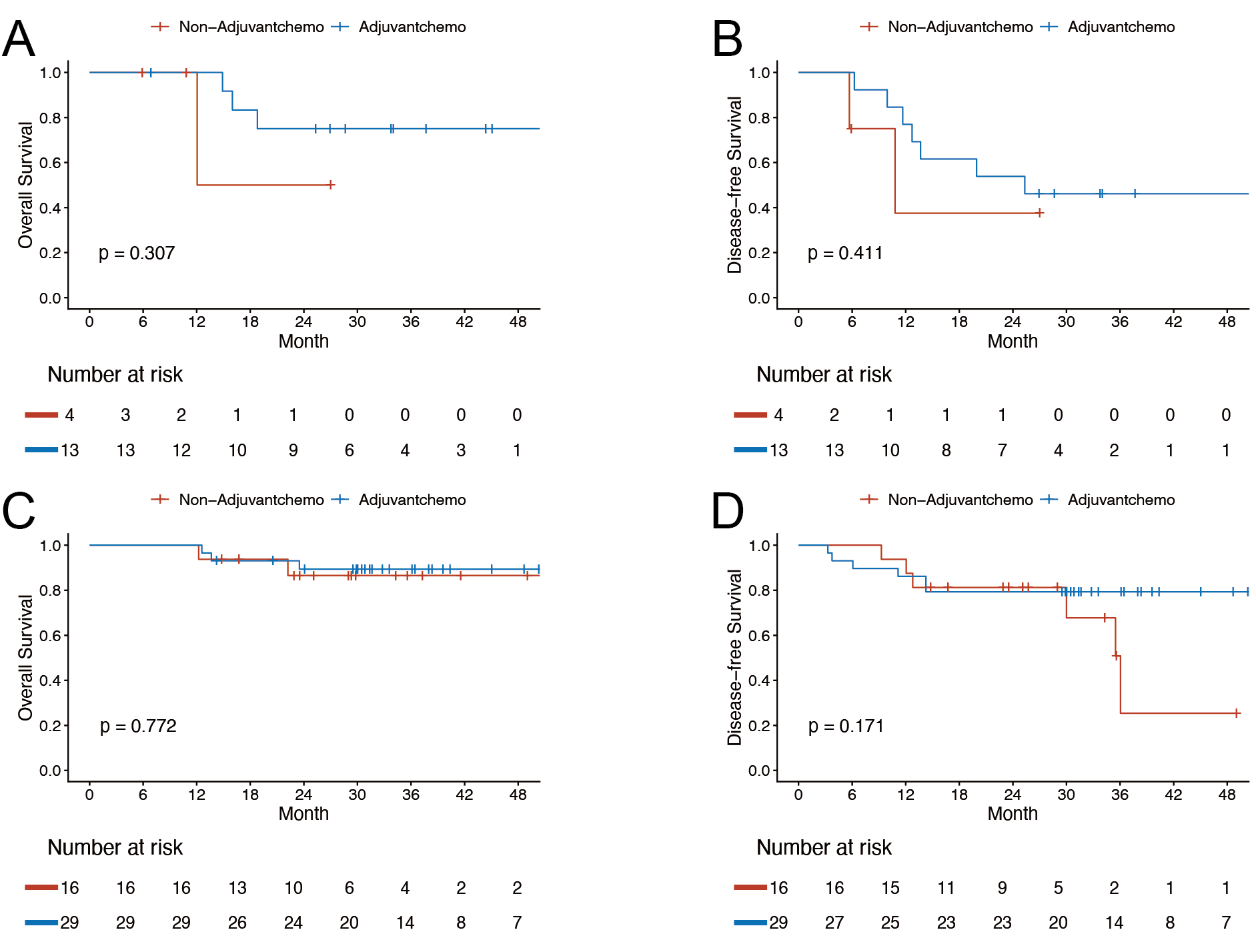

Supplement: Supplementary Figure 1 — Interobserver agreement for pathological assessment of ECE. Agreement between two independent pathologists was evaluated using Cohen’s kappa coefficient. [file SupplementaryFile1.zip › Supplementary Figure 6.PNG]

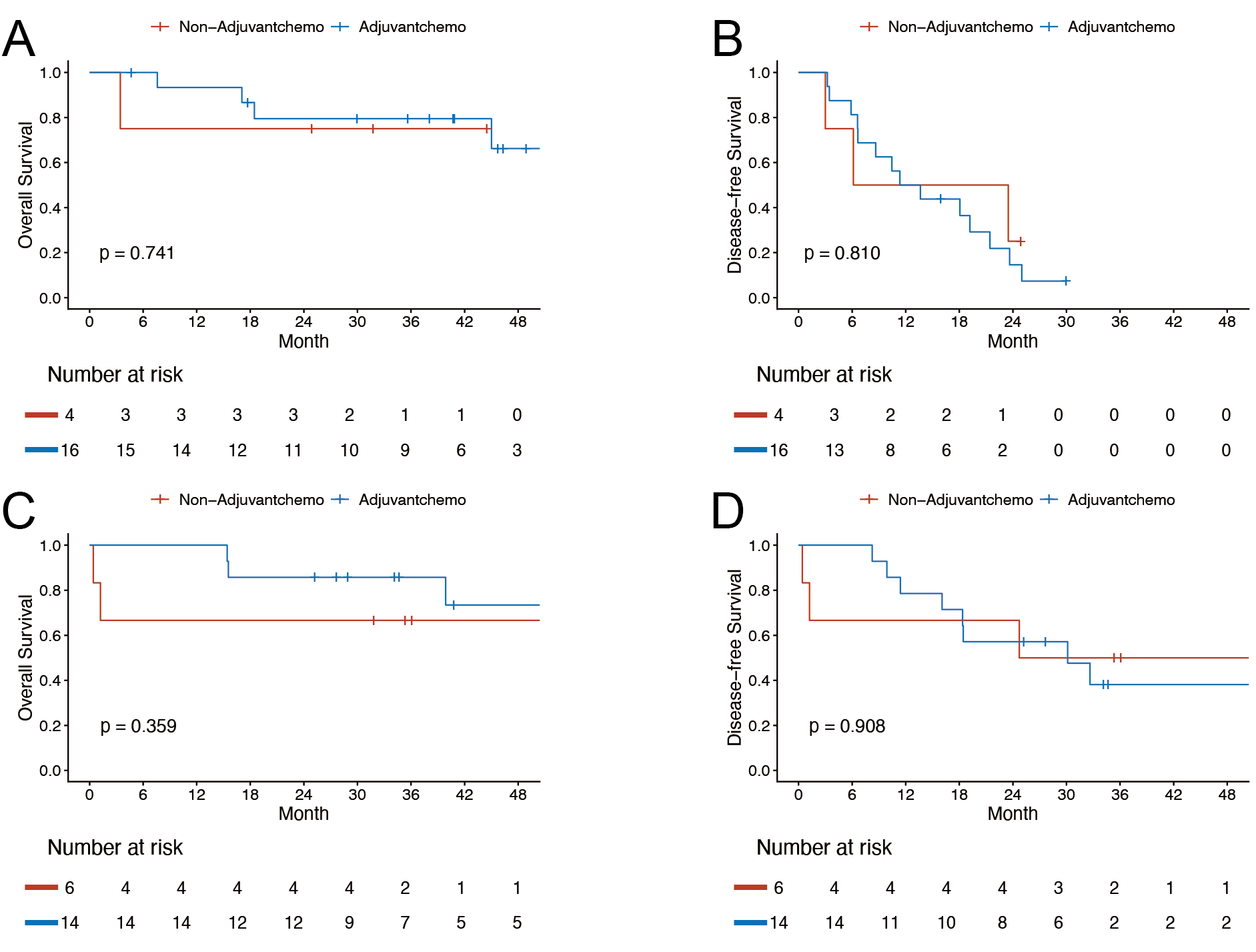

Supplement: Supplementary Figure 1 — Interobserver agreement for pathological assessment of ECE. Agreement between two independent pathologists was evaluated using Cohen’s kappa coefficient. [file SupplementaryFile1.zip › Supplementary Figure 7.PNG]

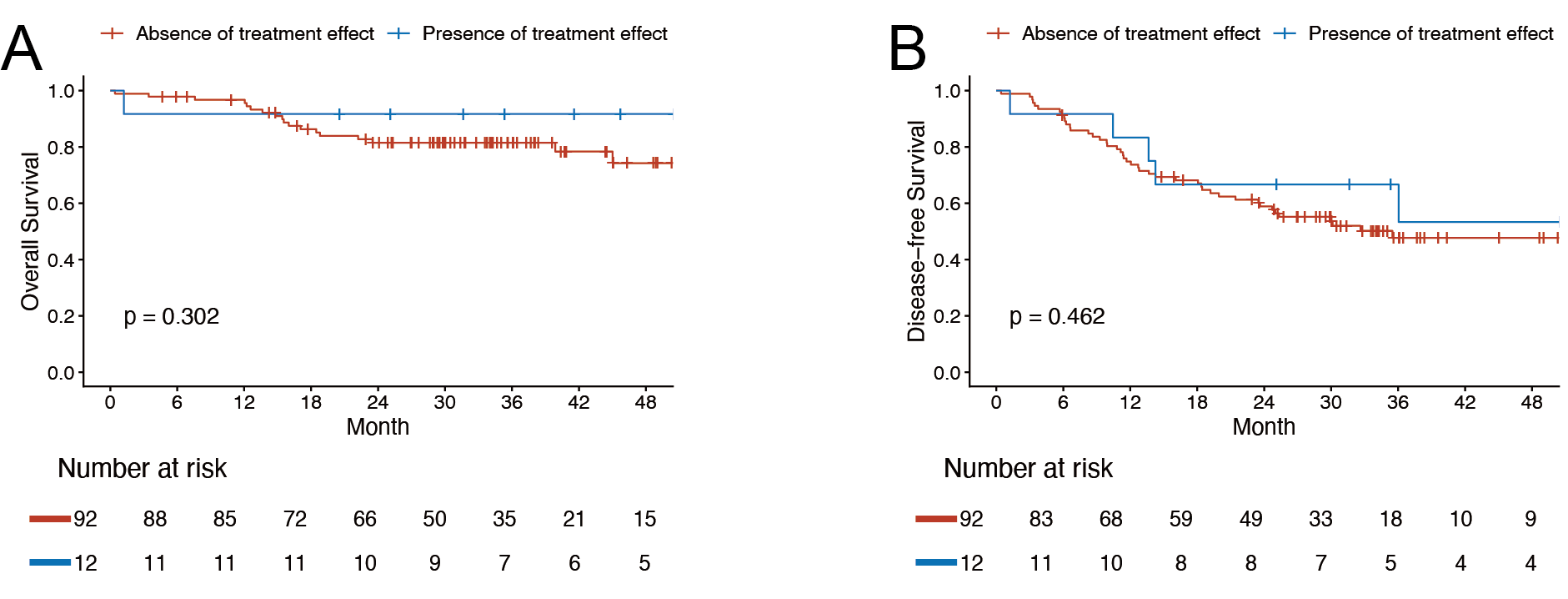

Supplement: Supplementary Figure 1 — Interobserver agreement for pathological assessment of ECE. Agreement between two independent pathologists was evaluated using Cohen’s kappa coefficient. [file SupplementaryFile1.zip › Supplementary Figure 8.PNG]

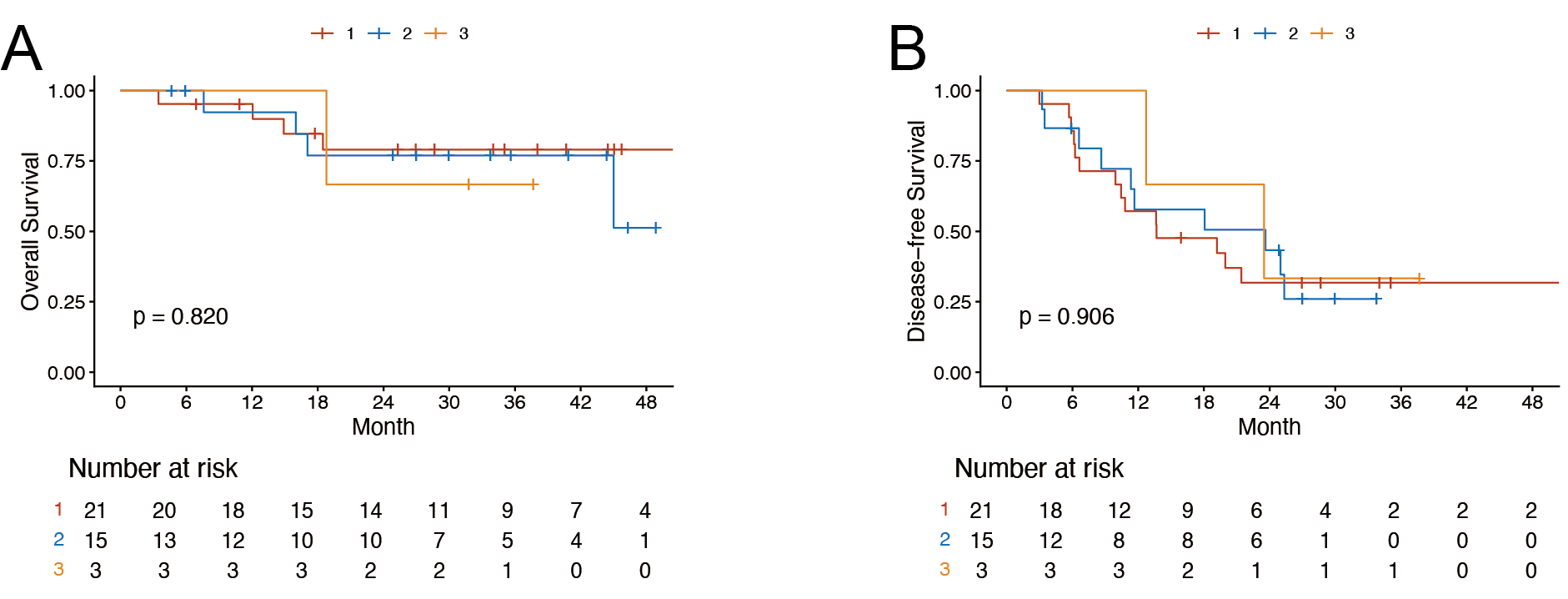

Supplement: Supplementary Figure 1 — Interobserver agreement for pathological assessment of ECE. Agreement between two independent pathologists was evaluated using Cohen’s kappa coefficient. [file SupplementaryFile1.zip › Supplementary Figure 9.PNG]

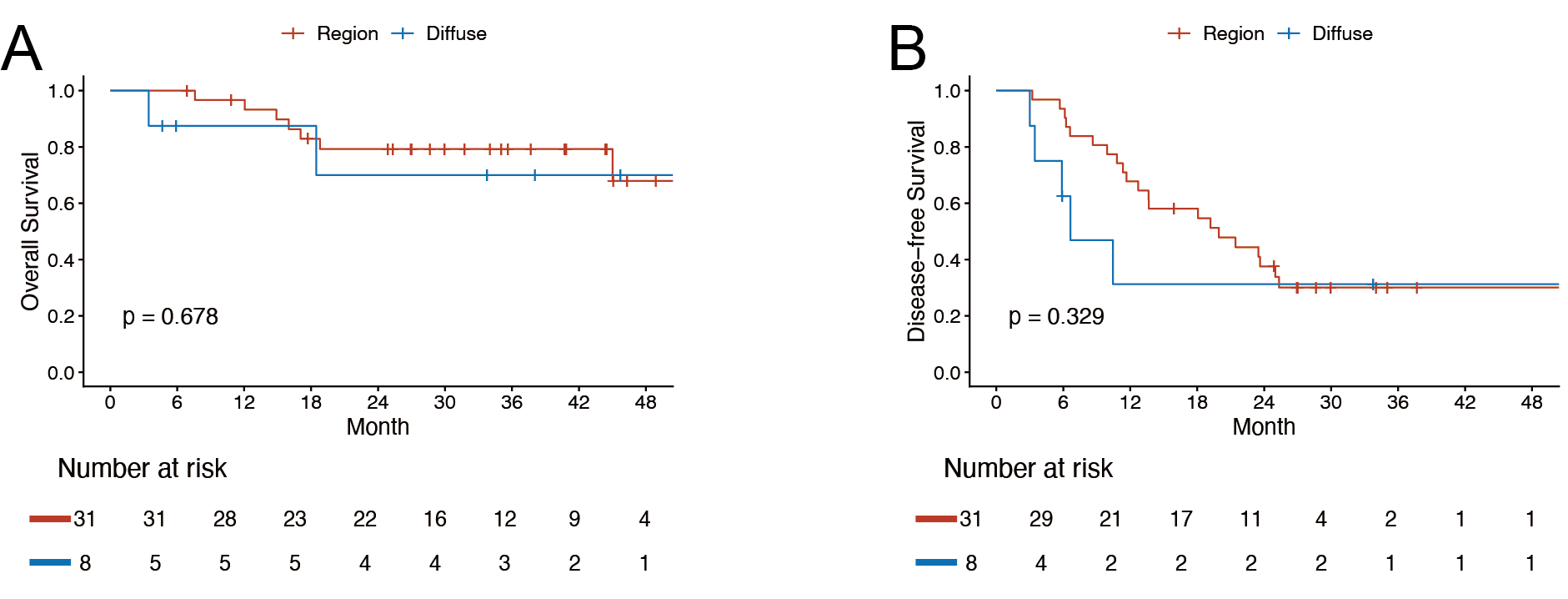

Supplement: Supplementary Figure 1 — Interobserver agreement for pathological assessment of ECE. Agreement between two independent pathologists was evaluated using Cohen’s kappa coefficient. [file SupplementaryFile1.zip › Supplementary Figure 10.PNG]

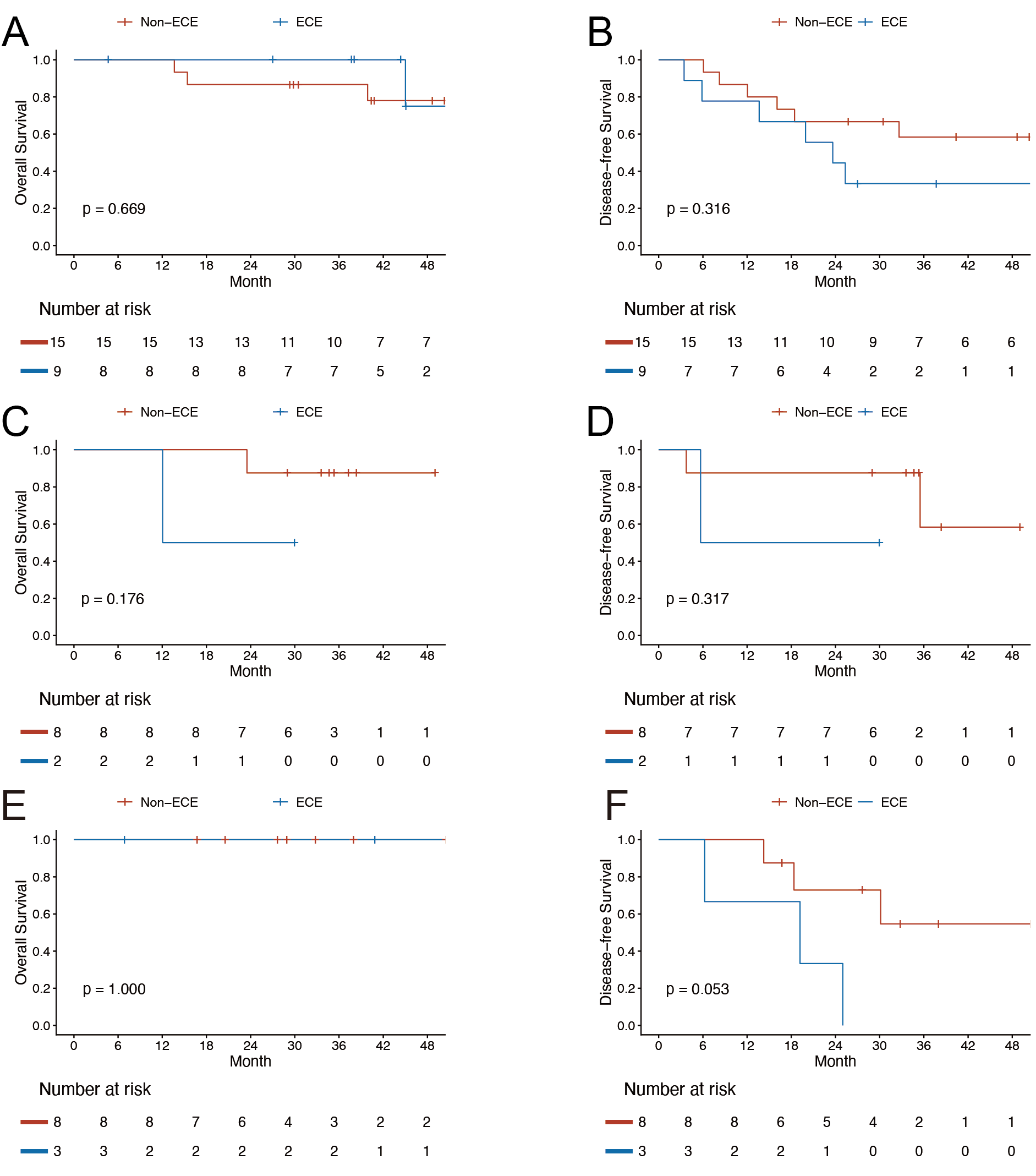

Supplement: Supplementary Figure 1 — Interobserver agreement for pathological assessment of ECE. Agreement between two independent pathologists was evaluated using Cohen’s kappa coefficient. [file SupplementaryFile1.zip › Supplementary Figure 12.PNG]

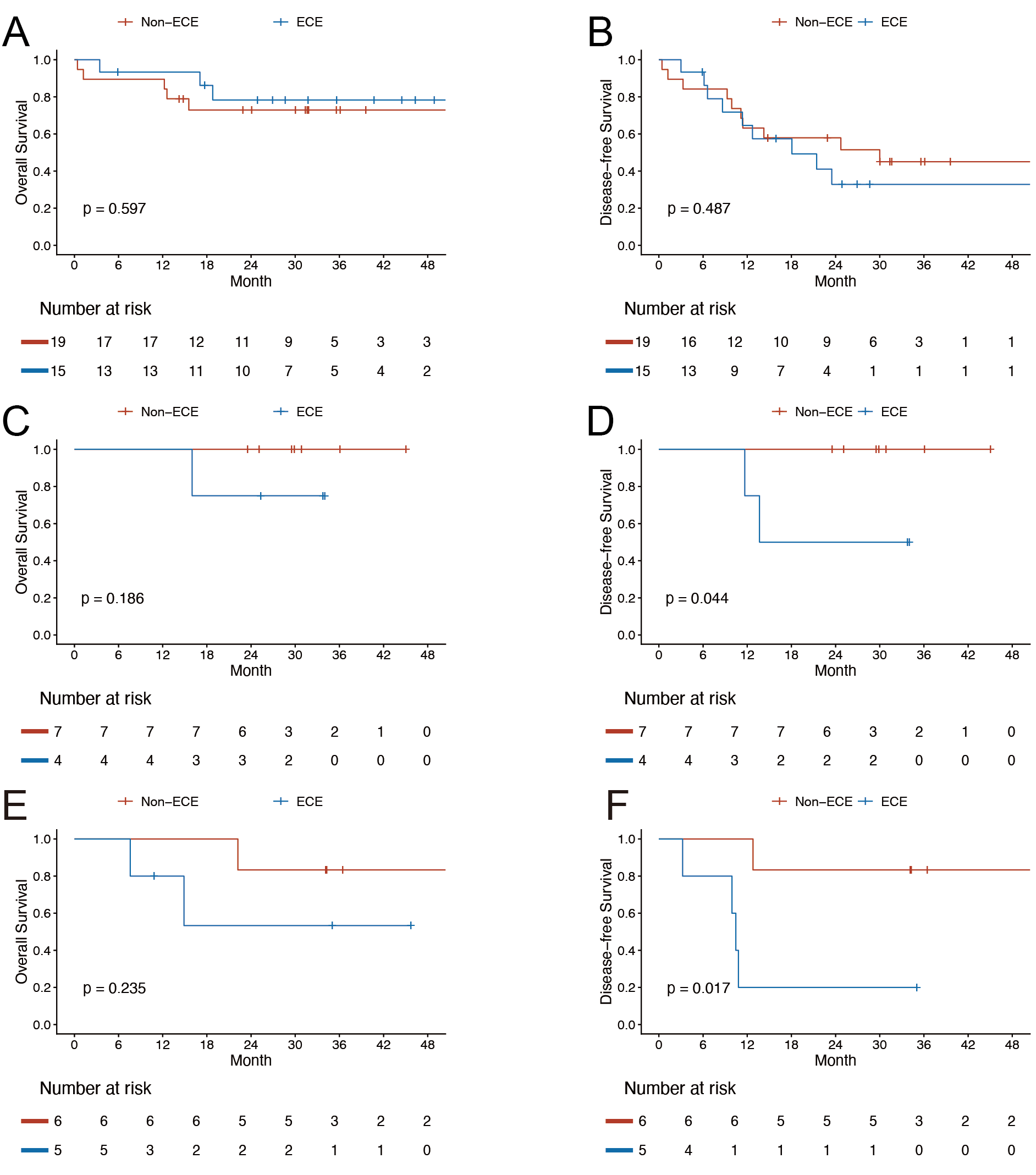

Supplement: Supplementary Figure 1 — Interobserver agreement for pathological assessment of ECE. Agreement between two independent pathologists was evaluated using Cohen’s kappa coefficient. [file SupplementaryFile1.zip › Supplementary Figure 13.PNG]

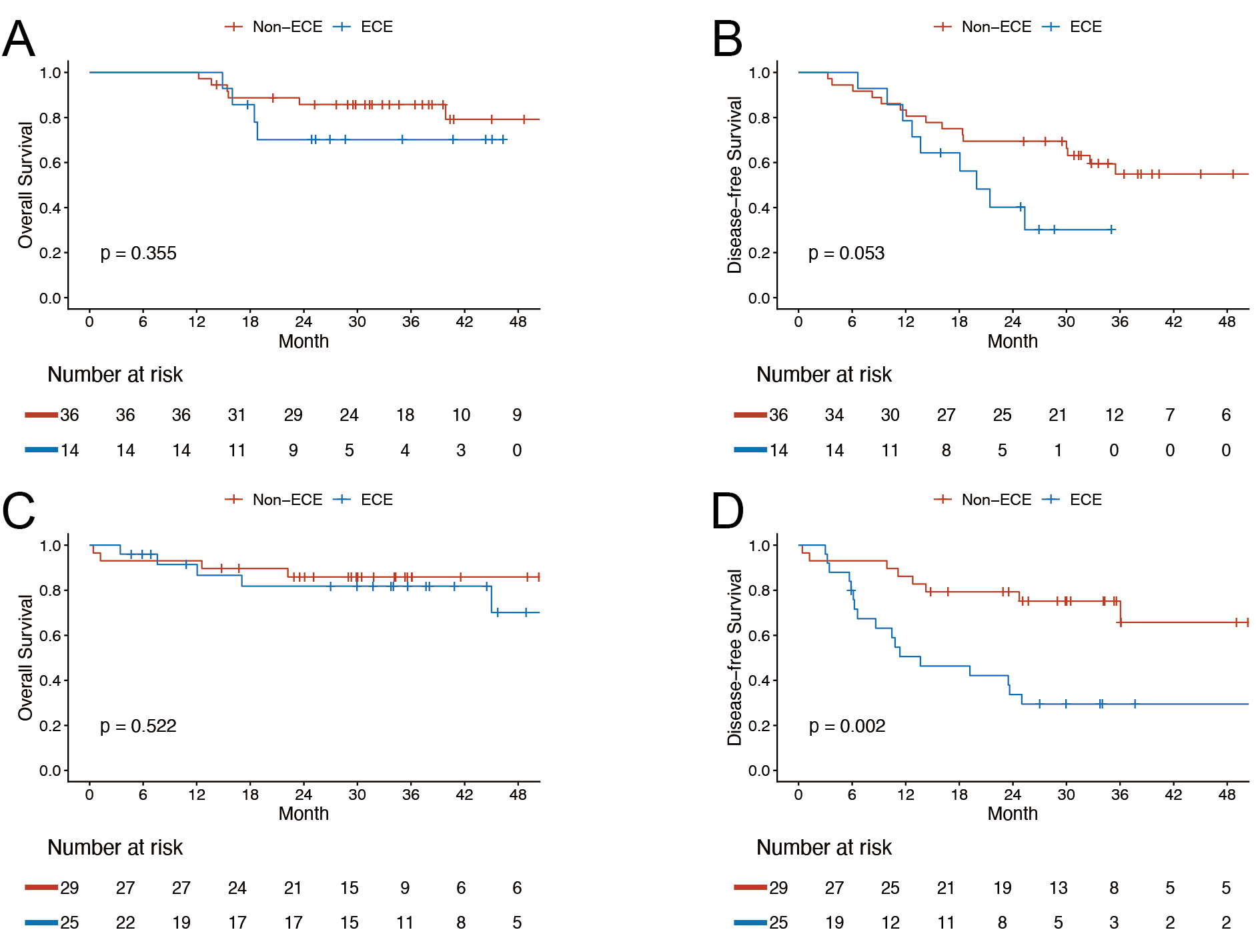

Supplement: Supplementary Figure 1 — Interobserver agreement for pathological assessment of ECE. Agreement between two independent pathologists was evaluated using Cohen’s kappa coefficient. [file SupplementaryFile1.zip › Supplementary Figure 14.PNG]

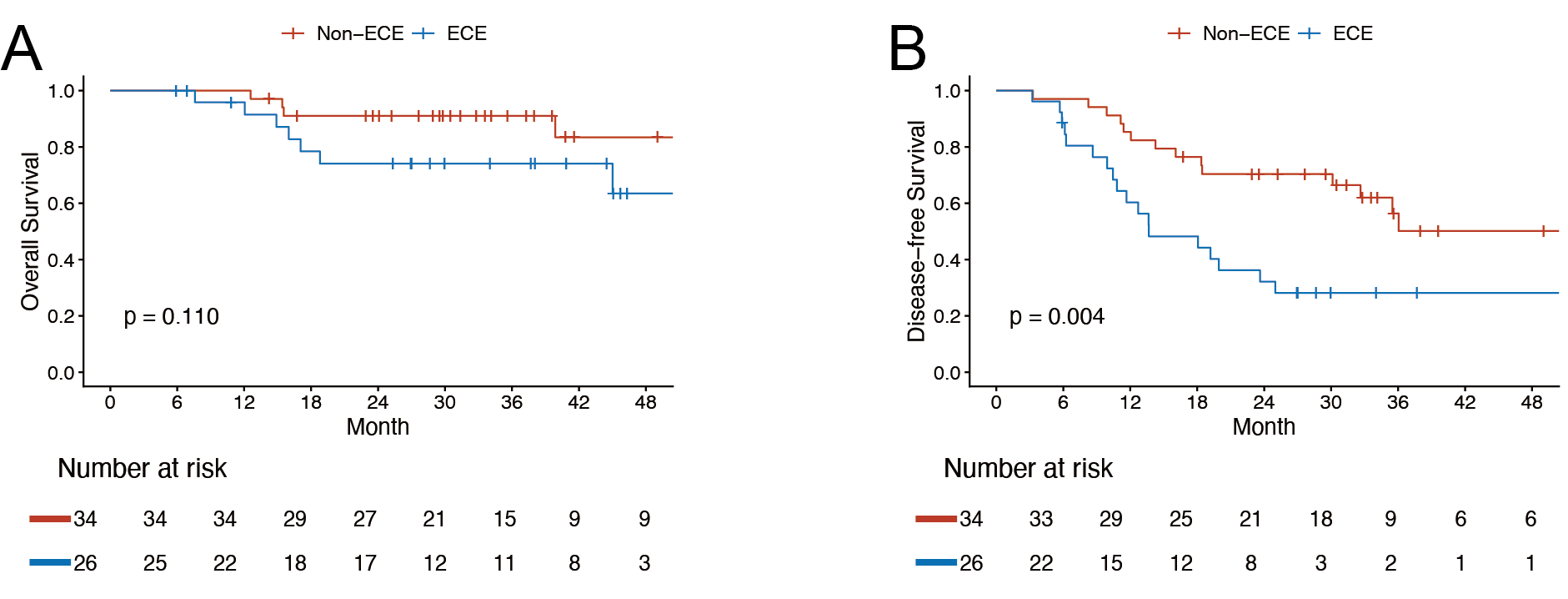

Supplement: Supplementary Figure 1 — Interobserver agreement for pathological assessment of ECE. Agreement between two independent pathologists was evaluated using Cohen’s kappa coefficient. [file SupplementaryFile1.zip › Supplementary Figure 16.PNG]

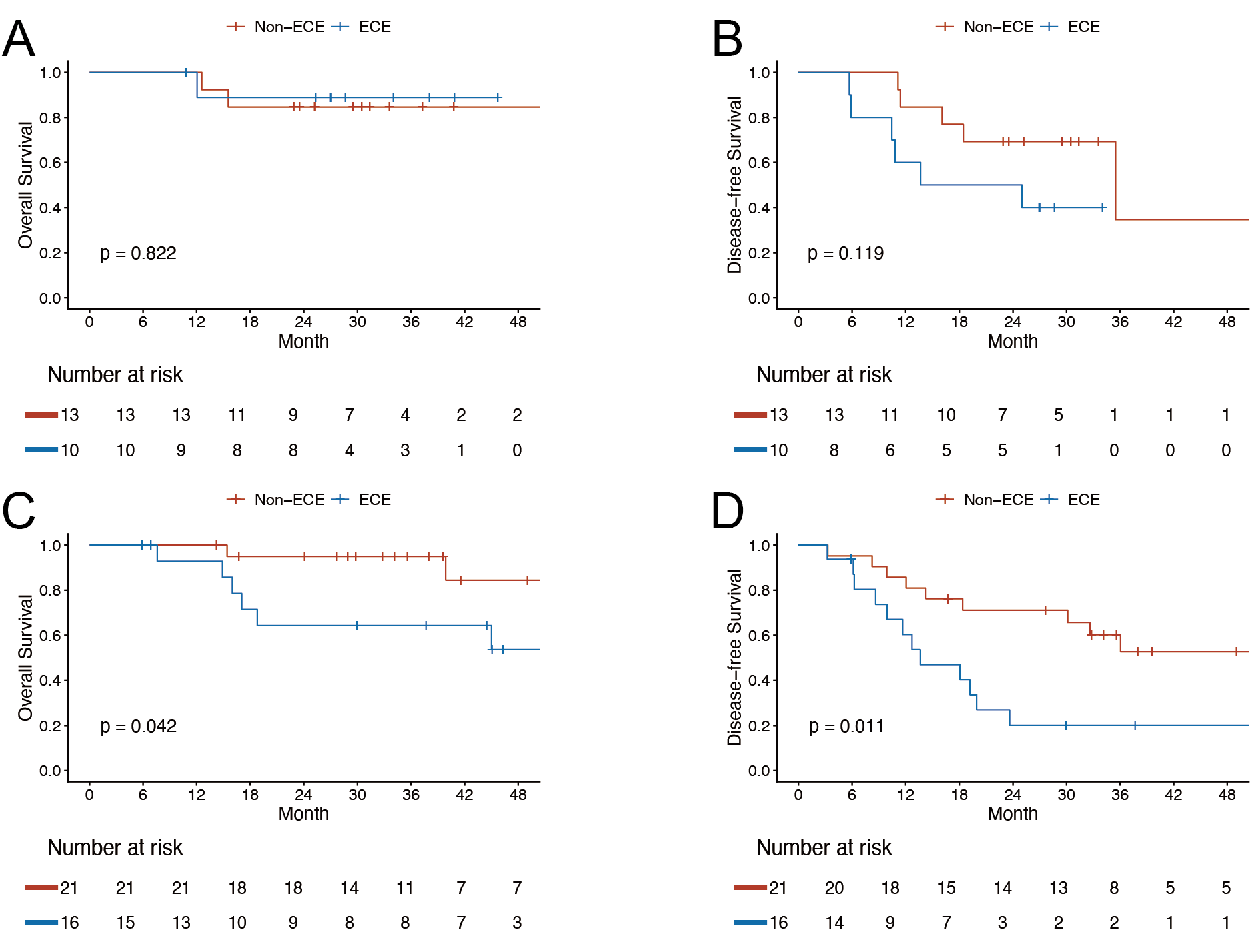

Supplement: Supplementary Figure 1 — Interobserver agreement for pathological assessment of ECE. Agreement between two independent pathologists was evaluated using Cohen’s kappa coefficient. [file SupplementaryFile1.zip › Supplementary Figure 17.PNG]

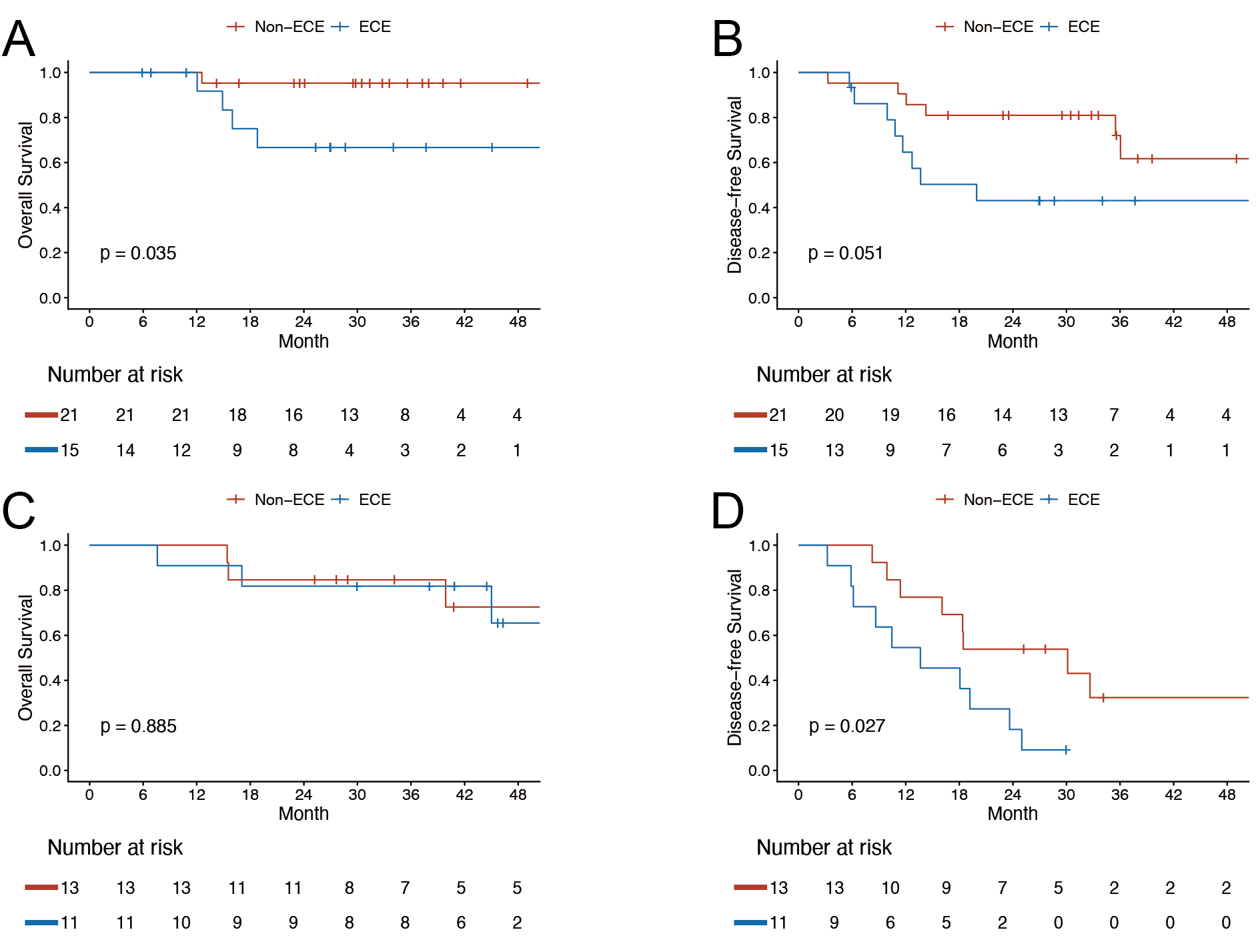

Supplement: Supplementary Figure 1 — Interobserver agreement for pathological assessment of ECE. Agreement between two independent pathologists was evaluated using Cohen’s kappa coefficient. [file SupplementaryFile1.zip › Supplementary Figure 18.PNG]

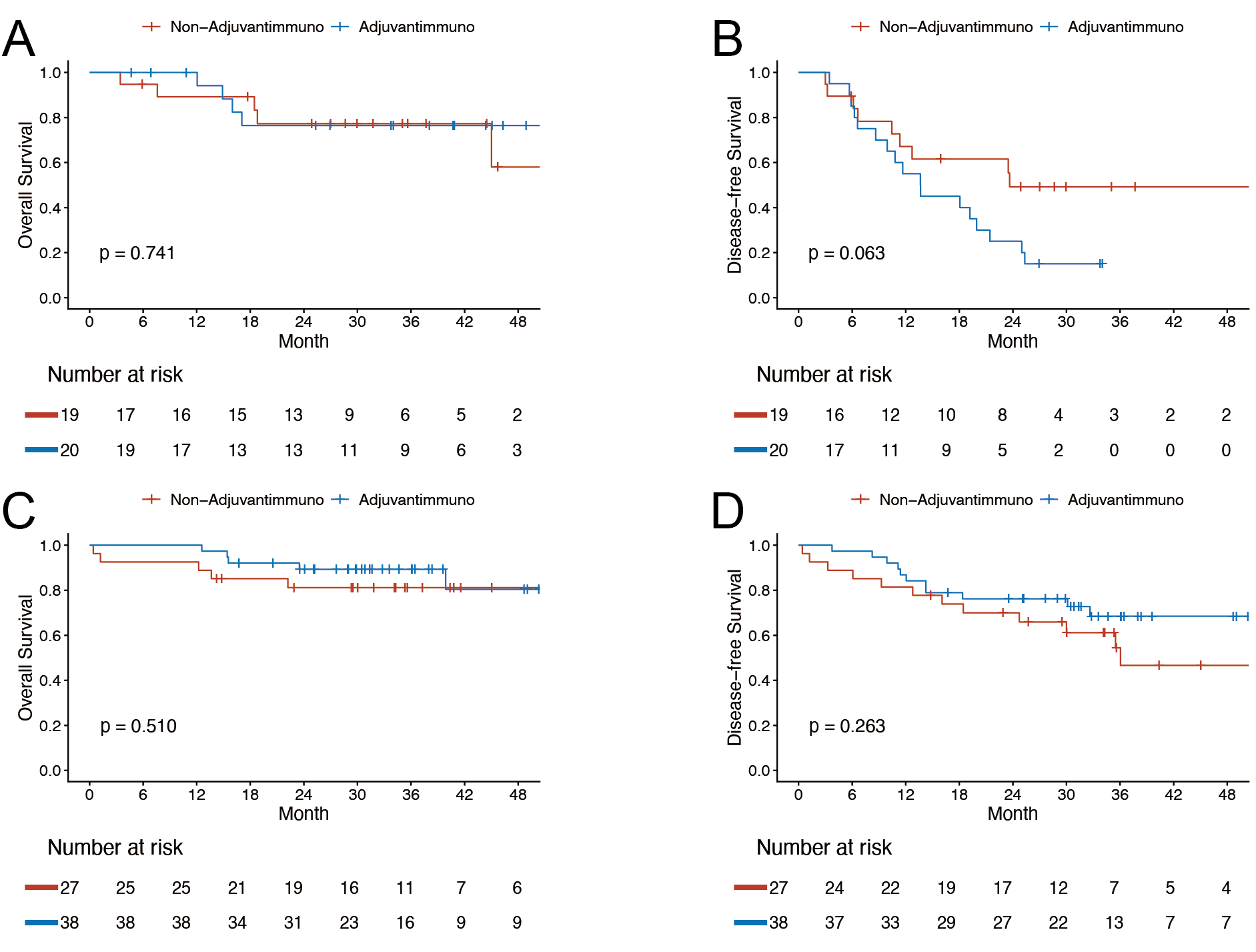

Supplement: Supplementary Figure 1 — Interobserver agreement for pathological assessment of ECE. Agreement between two independent pathologists was evaluated using Cohen’s kappa coefficient. [file SupplementaryFile1.zip › Supplementary Figure 2.PNG]

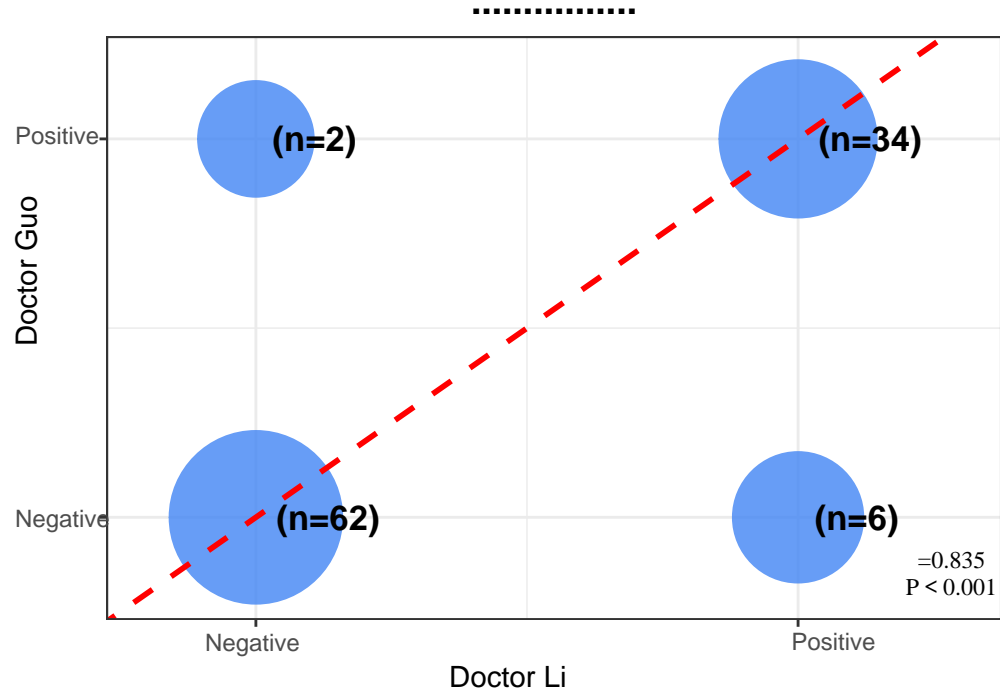

Supplement: Supplementary Figure 1 — Interobserver agreement for pathological assessment of ECE. Agreement between two independent pathologists was evaluated using Cohen’s kappa coefficient. [file SupplementaryFile1.zip › Supplementary Figure 1.PDF]

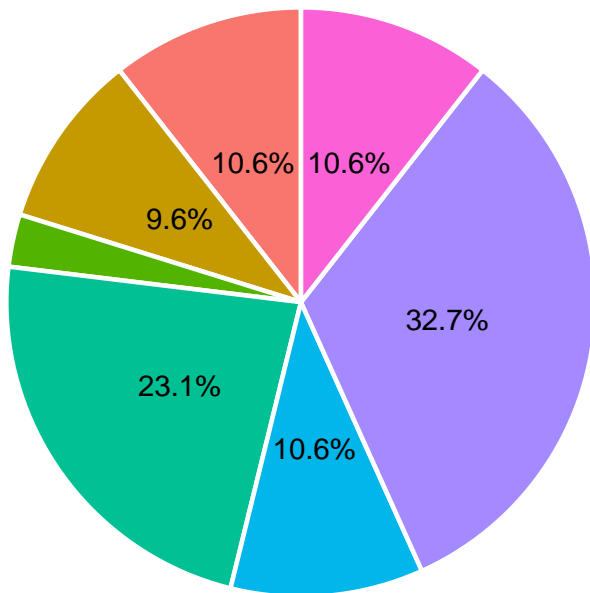

**Immunotherapy agent**

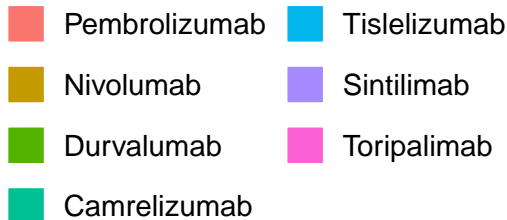

Supplement: Supplementary Figure 1 — Interobserver agreement for pathological assessment of ECE. Agreement between two independent pathologists was evaluated using Cohen’s kappa coefficient. [file SupplementaryFile1.zip › Supplementary Figure 11.PDF]

## SMD Before and After PSM

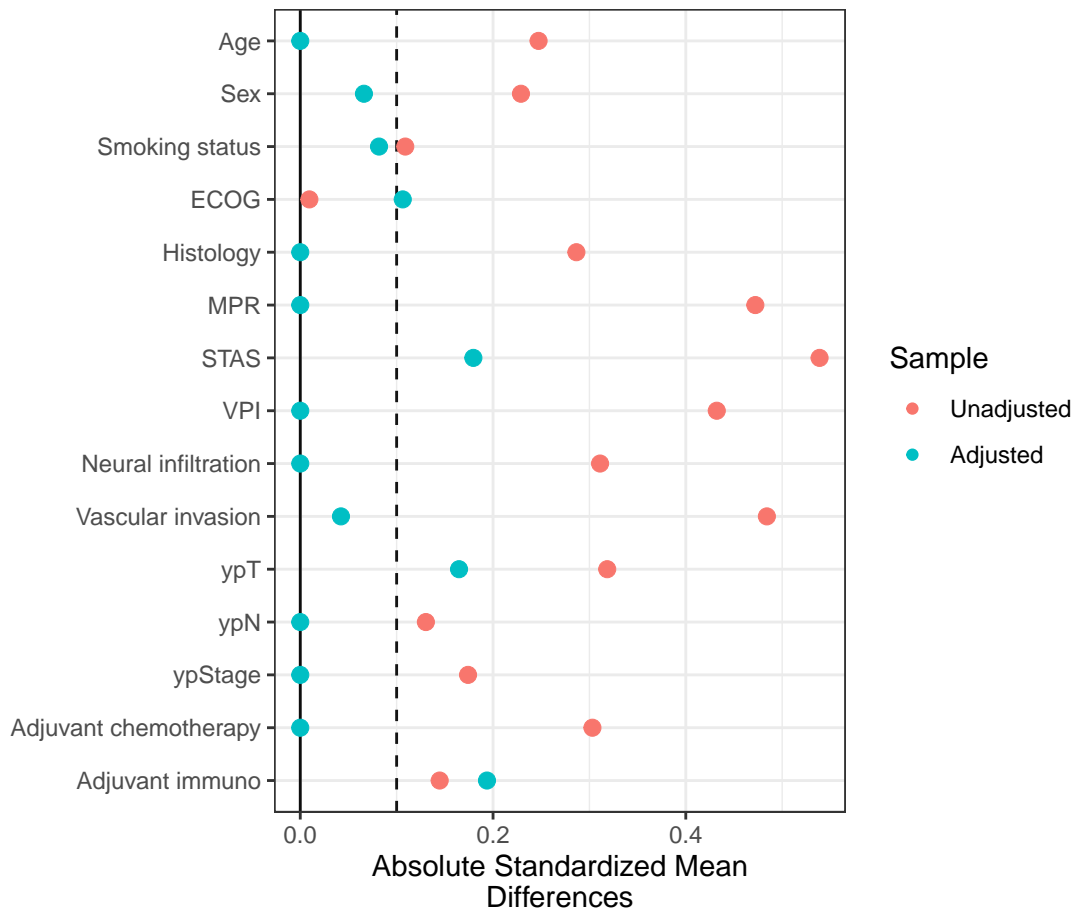

Supplement: Supplementary Figure 1 — Interobserver agreement for pathological assessment of ECE. Agreement between two independent pathologists was evaluated using Cohen’s kappa coefficient. [file SupplementaryFile1.zip › Supplementary Figure 15.PDF]

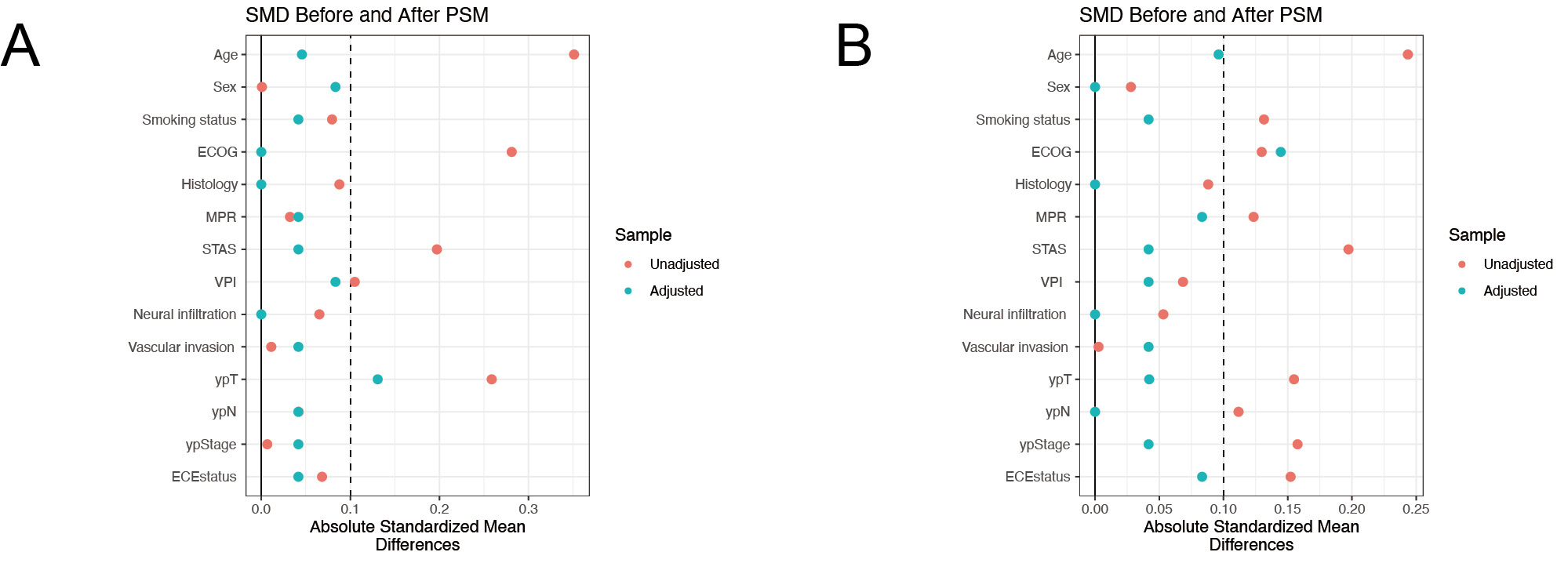

Supplement: Supplementary Figure 1 — Interobserver agreement for pathological assessment of ECE. Agreement between two independent pathologists was evaluated using Cohen’s kappa coefficient. [file SupplementaryFile1.zip › Supplementary Figure 19.JPEG]

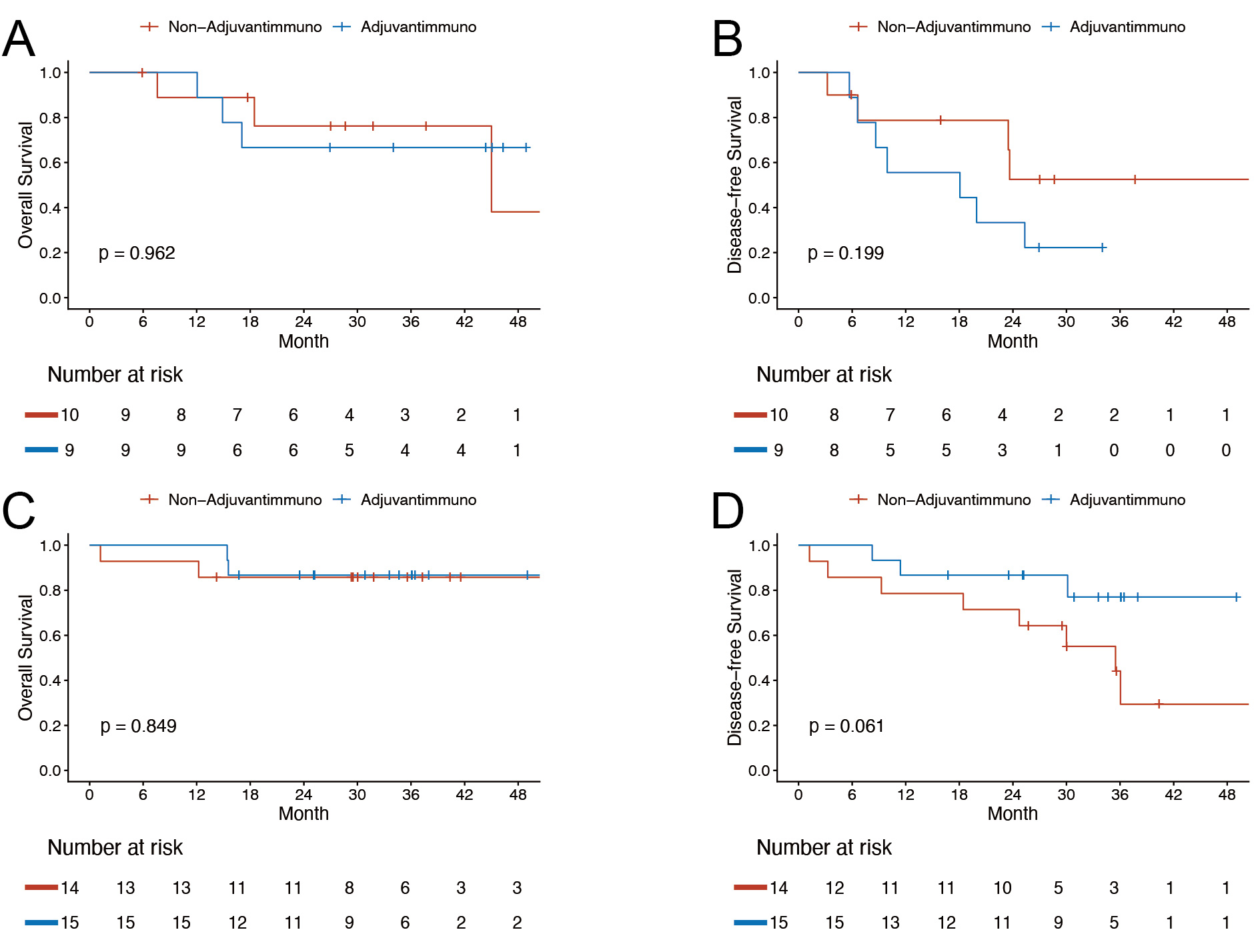

Supplement: Supplementary Figure 1 — Interobserver agreement for pathological assessment of ECE. Agreement between two independent pathologists was evaluated using Cohen’s kappa coefficient. [file SupplementaryFile1.zip › Supplementary Figure 20.JPEG]

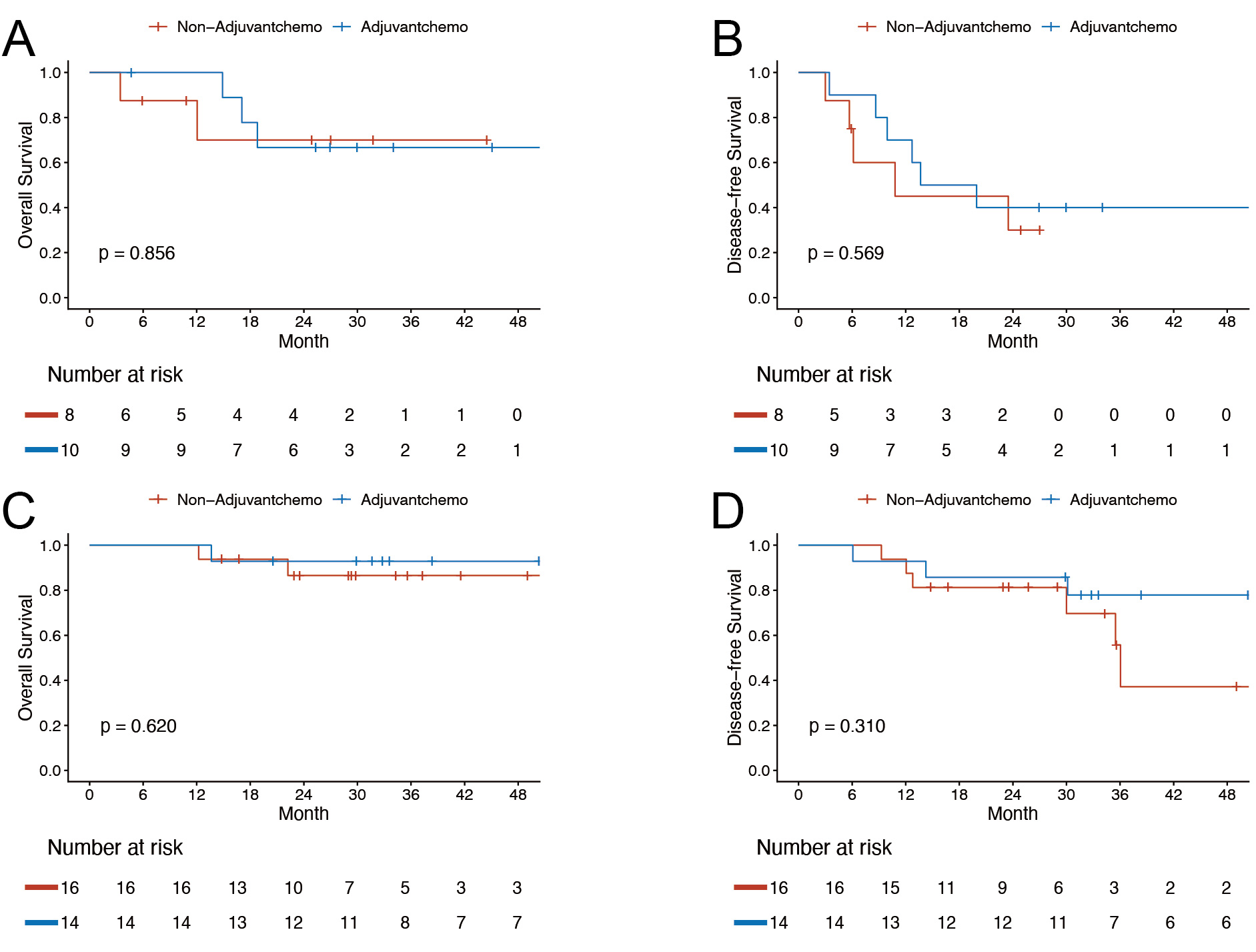

Supplement: Supplementary Figure 1 — Interobserver agreement for pathological assessment of ECE. Agreement between two independent pathologists was evaluated using Cohen’s kappa coefficient. [file SupplementaryFile1.zip › Supplementary Figure 21.JPEG]
